# Genetic and pharmacological inhibition of METTL3 alleviates renal fibrosis by reducing EVL m6A modification through an IGF2BP2‐dependent mechanism

**DOI:** 10.1002/ctm2.1359

**Published:** 2023-08-03

**Authors:** Wei‐Jian Ni, Hong Zhou, Hao Lu, Nan‐Nan Ma, Bing‐Bing Hou, Wei Li, Fan‐Xu Kong, Ju‐Tao Yu, Rui Hou, Juan Jin, Jia‐Gen Wen, Tao Zhang, Xiao‐Ming Meng

**Affiliations:** ^1^ Department of Pharmacy Anhui Provincial Hospital, The First Affiliated Hospital of USTC, Division of Life Sciences and Medicine University of Science and Technology of China Hefei Anhui People's Republic of China; ^2^ Inflammation and Immune Mediated Diseases Laboratory of Anhui Province Anhui Institute of Innovative Drugs School of Pharmacy Anhui Medical University Hefei Anhui People's Republic of China; ^3^ Department of Pharmacy Anhui Provincial Cancer Hospital, The First Affiliated Hospital of USTC, Division of Life Sciences and Medicine University of Science and Technology of China Hefei Anhui People's Republic of China; ^4^ Department of Urology The Second Affiliated Hospital of Anhui Medical University Hefei Anhui People's Republic of China; ^5^ Department of Urology The First Affiliated Hospital of Anhui Medical University Hefei Anhui People's Republic of China; ^6^ Department of Pharmacy The Second People's Hospital of Hefei Hefei Anhui People's Republic of China; ^7^ Research Center for Translational Medicine The Second Affiliated Hospital of Anhui Medical University Hefei Anhui People's Republic of China; ^8^ School of Basic Medicine Anhui Medical University Hefei Anhui People's Republic of China

**Keywords:** chronic kidney disease, insulin‐like growth factor 2 mRNA‐binding protein 2 (IGF2BP2), methyltransferase‐like 3 (METTL3), *N* ^6^‐methyladenosine (m6A), renal fibrosis, traditional Chinese medicine

## Abstract

**Background:**

*N* ^6^‐methyladenosine (m6A) is of great importance in renal physiology and disease progression, but its function and mechanism in renal fibrosis remain to be comprehensively and extensively explored. Hence, this study will explore the function and potential mechanism of critical regulator‐mediated m6A modification during renal fibrosis and thereby explore promising anti‐renal fibrosis agents.

**Methods:**

Renal tissues from humans and mice as well as HK‐2 cells were used as research subjects. The profiles of m6A modification and regulators in renal fibrosis were analysed at the protein and RNA levels using Western blotting, quantitative real‐time polymerase chain reaction and other methods. Methylation RNA immunoprecipitation sequencing and RNA sequencing coupled with methyltransferase‐like 3 (METTL3) conditional knockout were used to explore the function of METTL3 and potential targets. Gene silencing and overexpression combined with RNA immunoprecipitation were performed to investigate the underlying mechanism by which METTL3 regulates the Ena/VASP‐like (EVL) m6A modification that promotes renal fibrosis. Molecular docking and virtual screening with in vitro and in vivo experiments were applied to screen promising traditional Chinese medicine (TCM) monomers and explore their mechanism of regulating the METTL3/EVL m6A axis and anti‐renal fibrosis.

**Results:**

METTL3 and m6A modifications were hyperactivated in both the tubular region of fibrotic kidneys and HK‐2 cells. Upregulated METTL3 enhanced the m6A modification of EVL mRNA to improve its stability and expression in an insulin‐like growth factor 2 mRNA‐binding protein 2 (IGF2BP2)‐dependent manner. Highly expressed EVL binding to Smad7 abrogated the Smad7‐induced suppression of transforming growth factor‐β (TGF‐β1)/Smad3 signal transduction, which conversely facilitated renal fibrosis progression. Molecular docking and virtual screening based on the structure of METTL3 identified a TCM monomer named isoforsythiaside, which inhibited METTL3 activity together with the METTL3/EVL m6A axis to exert anti‐renal fibrosis effects.

**Conclusions:**

Collectively, the overactivated METTL3/EVL m6A axis is a potential target for renal fibrosis therapy, and the pharmacological inhibition of METTL3 activity by isoforsythiaside suggests that it is a promising anti‐renal fibrosis agent.

## Graphical abstract

1. Prefibrotic stimuli overactivate MELTT3 and m6A modifications in TECs and renal tissues.
2. Upregulated METTL3 enhances the m6A modification of EVL mRNA through an IGF2BP2‐dependent mechanism.
3. EVL binds to Smad7 to attenuate its inhibition of TGF‐β1/Smad3, which conversely promotes renal fibrosis.
4. Inhibition of METTL3 and the METTL3/EVL m6A axis markedly alleviates renal fibrosis.
5. Isoforsythiaside is a potential antifibrotic agent that inhibits the METTL3/EVL m6A axis.

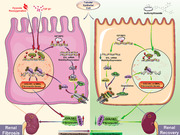

## 1 INTRODUCTION

Renal fibrosis represents the core pathogenesis and coexisting pathological phenotype in the development of almost all chronic kidney diseases (CKDs), such as obstructive nephropathy (ON), diabetic nephropathy (DN) and hypertensive nephropathy.^1^, ^2^ In parallel, renal fibrosis bridges the gap between acute kidney injury (AKI) and CKD.^3^ Thus, delaying or even preventing renal fibrosis would be the cornerstone of kidney disease management. Several studies have explored the dual function of transforming growth factor‐β (TGF‐β)/Smad signalling in the course of renal fibrosis.^4^, ^5^, ^6^ Moreover, research has also focused on the nonnegligible effects of the Wnt/β‐catenin and Hedgehog pathways in renal fibrosis.^7^, ^8^ Such findings have accelerated the advancement of mechanistic exploration and therapeutic translational research in renal fibrosis. Nevertheless, the complicated mechanisms of renal fibrosis remain unclear, and more intensive research efforts are urgently needed.

The importance of epigenetic modifications in the onset and evolution of numerous diseases cannot be disregarded.^9^ Specifically, *N* ^6^‐methyladenosine (m6A), as one of the most abundant and prevalent RNA epigenetic modifications, has attracted growing interest.^10^ Studies have demonstrated that the m6A modification is a reversible and dynamic incident coengaged and sustained by methyltransferases (m6A writers) and demethylases (m6A erasers), whereas reader proteins recognise m6A modifications.^11^, ^12^ Throughout the process, methyltransferase‐like 3 (METTL3) acts as the catalytic core to catalyse and activate the m6A modification with the structural support of METTL14 and the stabilisation of Wilms’ tumour 1‐associating protein (WTAP), while demethylases alkB homologue 5 (ALKBH5) and fat mass and obesity‐associated protein (FTO) are responsible for erasing and reversing the modifications.^13^ Functional studies have shown that diverse and potent m6A modifications govern the entire life cycle of mammalian RNAs, mainly including but not limited to mRNA splicing, translation, decay and degradation, thereby affecting gene expression, protein production and corresponding biological functions, such as cell differentiation and embryonic development.^14^, ^15^, ^16^ In recent studies, the widespread presence of m6A modifications has been confirmed in the human kidney.^17^, ^18^ In parallel, our prior investigations have reported the critical roles of methyltransferase, demethylase and reader protein‐mediated m6A modifications in renal diseases such as AKI,^19^ DN^20^ and alcohol‐induced kidney injury,^21^ but their elaborated functions and mechanisms in renal fibrosis remain uncertain and need to be thoroughly explored through extensive studies.

To evaluate the influence of METTL3‐dominated m6A modification on renal fibrosis, we performed relevant experimental studies using *Ksp‐Cre*‐METTL3 (*Flox/Flox*) conditional knockout (cKO) mice and small interfering RNA (siRNA)‐silenced renal tubular epithelial cells (HK‐2). By performing RNA sequencing (RNA‐seq) and methylation RNA immunoprecipitation sequencing (MeRIP‐seq) on tubular regions of mouse kidney tissues, we investigated potential targets of METTL3‐mediated m6A modification and the detailed mechanisms of action. Subsequently, we investigated the function and possible mechanism of the potential target termed Ena/VASP‐like (EVL) within the renal fibrosis process.

Although investigations have highlighted the prominent function of METTL3‐mediated m6A modifications in diseases, therapeutic agents targeting METTL3 have not been thoroughly explored.^19^, ^22^ Given the variable medicinal properties of traditional Chinese medicine (TCM) monomers, such as antioxidant, anticancer and antibacterial effects, they have emerged as an important resource for novel drug development.^23^, ^24^, ^25^ On this basis, we explored potential anti‐renal fibrosis agents that target METTL3 and investigated their functions through molecular docking combined with high‐throughput drug virtual screening as well as pharmacological studies based on the structure of the METTL3 protein and the database of TCM monomers. After a comprehensive screening, we identified and validated a novel TCM monomer inhibitor of METTL3 at the pharmacological level and evaluated its effectiveness in treating renal fibrosis in mice.

Together, the present findings provide insight into the function and mechanism of METTL3‐mediated EVL m6A modifications in renal fibrosis and comprehensively expand the understanding of related studies. This study identified a novel TCM monomer inhibitor and provided evidence suggesting that it may be a valuable therapeutic strategy for targeting METTL3‐EVL m6A axis‐mediated renal fibrosis.

## 2 MATERIALS AND METHODS

### 2.1 Reagents and chemicals

Recombinant human TGF‐β1 (human cell/HEK293‐derived) was acquired from PeproTech and R&D Systems. Isoforsythiaside was provided by TargetMol Biotechnology and Yuanye Biotechnology (B21536). All reagents and antibodies involved in this study were obtained from commercial sources and are listed in Table S1.

### 2.2 Human specimens

Human fibrotic kidney specimens were obtained from patients undergoing nephrectomy for nonfunctional kidneys due to hydronephrosis or obstruction in the Second Affiliated Hospital of Anhui Medical University. Paracancerous tissues from patients with renal cell carcinoma (RCC) were used as controls. The clinical profile of controls and ON patients with fibrotic kidneys is presented in Table 1. The related studies were authorised by the Biomedical Ethics Committee of Anhui Medical University (Ethical Approval No. 83220413) and were implemented with the subjects' written consent and understanding.

**TABLE 1:** Clinical features of patients with renal fibrosis in this study.

Patients	Gender	Age (years)	Specimen origin	Pathologic diagnosis	Comorbidity
Control1	Male	55	RCC, paraneoplastic tissue	Renal clear cell carcinoma	No
Control2	Male	57	RCC, paraneoplastic tissue	RCC	No
Control3	Male	36	RCC, paraneoplastic tissue	Renal clear cell carcinoma	No
Control4	Male	47	RCC, paraneoplastic tissue	RCC	No
Control5	Male	59	RCC, paraneoplastic tissue	RCC	No
Control6	Male	65	RCC, paraneoplastic tissue	RCC	No
ON Patient1	Male	58	Fibrotic kidney tissue	ON/hydronephrosis, nonfunctional kidney	No
ON Patient2	Male	71	Fibrotic kidney tissue	ON/hydronephrosis, nonfunctional kidney	No
ON Patient3	Male	50	Fibrotic kidney tissue	ON/hydronephrosis, nonfunctional kidney	No
ON Patient4	Male	36	Fibrotic kidney tissue	ON/hydronephrosis, nonfunctional kidney	No
ON Patient5	Male	57	Fibrotic kidney tissue	ON/hydronephrosis, nonfunctional kidney	No
ON Patient6	Male	58	Fibrotic kidney tissue	ON/hydronephrosis, nonfunctional kidney	No

Abbreviations: ON, obstructive nephropathy; RCC, renal cell carcinoma.

### 2.3 Animal studies

C57BL/6J mice (male, 20−22 g, 6−8 weeks) were recruited from the Animal Experimentation Center of Anhui Medical University (Nos. 202101393, 202102844 and 20220123). Mice were acclimatised to a standard chow diet for 2 weeks prior to the start of the study under conditions of constant room temperature and a light–dark cycle (12 h–12 h). All animal experiments were authorised by the Animal Experimentation Ethics Committee of Anhui Medical University (No. LLSC20211525) and conducted following the Guide for the Care and Use of Laboratory Animals. For the unilateral ureteral obstruction (UUO)‐induced ON model, mice were subjected to permanent ureteral ligation of the left kidney ureter under aseptic and anaesthetic conditions, whereas sham‐operated mice merely lacked ligation under equivalent conditions. The mice were sacrificed under anaesthesia at 3, 7 and 14 days postoperatively. For the ischemia‒reperfusion (I/R)‐induced renal fibrosis model, mice were recovered with blood supply after 42 min of bilateral renal artery clamping under both aseptic and anaesthetic conditions. These mice were sacrificed under anaesthesia at 7 and 14 days postoperatively. For drug treatment, isoforsythiaside was injected intraperitoneally at concentrations of 10, 25 and 50 mg/kg per day when the model was established. Similarly, the sham‐operated group received an equivalent volume of saline intraperitoneally as a control. The harvested kidneys have been primarily used for morphological and histopathological observations and molecular biology studies.

### 2.4 Cell lines and culture conditions

The STR‐identified human renal tubular epithelial cell line (HK‐2) was supplied by Procell Technology. Dulbecco's modified Eagle medium/F12 medium with 10% foetal bovine serum (FBS) was used for regular cell cultures under 37°C, 5% CO_2_ and 95% air conditions. Before adding TGF‐β1 (5 ng/mL) to stimulate cells, the FBS concentration was reduced to .5% overnight starvation. The hypoxia–reoxygenation (H/R)‐induced AKI‐to‐CKD model of HK‐2 cells consisted of three consecutive cycles of H/R treatment (9 h hypoxia/3 h reoxygenation).

### 2.5 Overexpression and knockdown of genes

Cells were transfected with LipoFiter 3.0 (Hanbio) for overexpression plasmids (Hanbio) or siRNA (GenePharma Co. Ltd.). Following transfection for 6−8 h, the culture was continued for 24−48 h with fresh medium. Subsequently, the cells were treated with TGF‐β1 (5 ng/mL) and/or candidate compounds and harvested at specific times (12, 24 and 30 h). Total RNA and protein were extracted for analysis. Selected sequences are listed in Table 2.

**TABLE 2:** Primers and RNA sequences used in this study.

Terms	Forward primer (5′−3′)	Reverse primer (5′−3′)
Primers for qRT‒PCR (mouse)		
METTL3	CCCAACCTTCCGTAGTGATAG	TGGCGTAGAGATGGCAAGAC
Acta1	CCCAAAGCTAACCGGGAGAAG	GACAGCACCGCCTGGATAG
Ccdc9	AGGAGACAACTCAACCTCTGAT	GCGCTCATATTCGGCAATCTTC
Ckap5	TGATTCCAATGCAGTGGTTCAA	ACCTGATACAACCTCTCCCGT
Ect2	GGCCTTAAAGGAAATGAAAGTGC	ACCAGGTTCAGCATACTCGTA
Evl	GGGATTCAGCCGGATCAACAT	CTGGTCCTGTAGCTTGACCC
Frem1	CCTGAGGGTTGCAGTCCCTA	CATCCAGAATTGGGCATCCAT
Igf1	CACATCATGTCGTCTTCACACC	GGAAGCAACACTCATCCACAATG
Itgal	CCAGACTTTTGCTACTGGGAC	GCTTGTTCGGCAGTGATAGAG
Lims	ATCGTGAACAGTAATGGTGAGC	CACATCGGAAGCACTCAGGAT
Lsp1	AGCTGCTGAGGCTCACAAC	CAGGCTGATGAGTGTCTGCTG
Nfkb1	ATGGCAGACGATGATCCCTAC	CGGAATCGAAATCCCCTCTGTT
Npy6r	GCAAGAGCAACAACTCGGC	CGTAAAAGGGATGCACATGACA
Pglyrp2	CCGACGGCTATCTGTACCAG	CAGTGACCCAGTGTAGTTGC
Rad51	GTCCACAGCCTATTTCACGGT	ACAGCCTCCACTGTATGGTAAC
Rassf4	AAGCCTGCTCAAAACCTACAAC	GGATGGGTCGTCTGAGTCC
Serpinb6c	AGGAACCACTGCGATCCAGAT	GTCTTGTTCACTTCGCTGAGA
Tceal5	GAGAGCAAGCCCGATTCCC	TTCCGGGGCACATAATCTTCA
Vgf	AAGGATGACGGCGTACCAGA	TGCCTGCAACAGTACCGAG
Zc3hav1	CCCGAAGCGCAACTCTACG	CGCTGGGACTGTGCATAGTG
COL1A1	GCTCCTCTTAGGGGCCACT	CCACGTCTCACCATTGGGG
αSMA	CGGGCTTTGCTGGTGATG	CCCTCGATGGATGGGAAA
Fibronectin	CTAGGCAATGCGTTGGTTTGTA	ACGCTCATAAGTGTCACCCACTC
β‐Actin	CATTGCTGACAGGATGCAGAA	ATGGTGCTAGGAGCCAGAGC
Primers for qRT‒PCR (human)		
METTL3	GCCTTTGCCAGTTCGTTAGT	TGACCTTCTTGCTCTGTTGTTC
METTL14	AAATGCTGGACTTGGGATGA	TGAGGCAGTGTTCCTTTGTTC
WTAP	TGCCCAACTGAGATCAACAA	CATTCGACACTTCGCCATTA
FTO	GGTTGATAAGGCACAAGGCA	TCAGCAGGTAATGTTCGGGC
ALKBH5	GCAGAGTTGTTCAGGTTGCC	GTCAGGACCACTGCACTAGC
VIRMA	CTTGGCAAGTGGCTTGAACC	ACGTAAGGCAGTGGTAAGGC
Acta1	GGCATTCACGAGACCACCTAC	CGACATGACGTTGTTGGCATAC
Ccdc9	TCTCTGACCGTAAATCCAAGGA	CTGGTTGCGCTCATACTCG
Ckap5	TGTGGAAAGCAAGGTTAAGTGG	ACTCTGGGCTCTTTTCATCCT
Ect2	ACTACTGGGAGGACTAGCTTG	CACTCTTGTTTCAATCTGAGGCA
Evl	CTTCCGTGATGGTCTACGATG	TGCAACTTGACTCCAACGACT
Frem1	GCCTGTGGTAACCAGGAACAA	CGCAGGTGTATCAGGGTCG
Igf1	GCTCTTCAGTTCGTGTGTGGA	GCCTCCTTAGATCACAGCTCC
Itgal	GCTTATCATCATCACGGATGG	CTCTCCTTGGTCTGAAAATGCT
Lims	TGGCCGAGTTATCAAAGCCAT	CTTTCTCACGATTATGACAGGGG
Lsp1	GGAGCACCAGAAATGTCAGCA	TCGGTCCTGTCGATGAGTTTG
Nfkb1	AACAGAGAGGATTTCGTTTCCG	TTTGACCTGAGGGTAAGACTTCT
Npy6r	AACCACCCAGCATCTAATACAAC	GCCCACAATTAAGACCACAGT
Pglyrp2	TCCTACTCGGATTGCTACTGTG	AAGTGGTAGAGGCGATTGTGG
Rad51	CAACCCATTTCACGGTTAGAGC	TTCTTTGGCGCATAGGCAACA
Rassf4	CCTGCTGAAAACCTACAACTGC	GGCTGTGATGTTCCCGTTC
Serpinb6c	AGCACATCAACACCTGGGTA	CTCCCTGGTGTCCTCTTTGT
Tceal5	GAGGGAAAGCGAGAGGATGA	CTTTGTGGCTTGTCCTCACC
Vgf	GGAACTGCGAGATTTCAGTCC	GTGCGGGTTTCCGTCTCTG
Zc3hav1	CCGGTGCAACTATTCGCAGT	TCAGTCCAGAGAGTTCGTGATTT
COL1A1	CGGACGACCTGGTGAGAGA	CATTGTGTCCCCTAATGCCTT
αSMA	ATCCGATAGAACACGGCATC	AGTCACGCCATCTCCAGAGT
Fibronectin	CTAGGCAATGCGTTGGTTTGTA	ACGCTCATAAGTGTCACCCACTC
GAPDH	GCAAGTTCAACGGCAGCA	CGCCAGTAGACTCCACGAC

siRNA	Forward primer (5′−3′)	Reverse primer (5′−3′)
siRNA sequences used for gene knockdown		
si‐METTL3#1	GCUCAACAUACCCGUACUATT	UAGUACGGGUAUGUUGAGCTT
si‐METTL3#2	GCAGAACAGGACUCGACUATT	UAGUCGAGUCCUGUUCUGCTT
si‐IGF2BP1#1	CCTGGCCCATAATAACTTT	AAAGTTATTATGGGCCAGG
si‐IGF2BP1#2	GCTCCCTATAGCTCCTTTA	TAAAGGAGCTATAGGGAGC
si‐IGF2BP2#1	GCTGTTAACCAACAAGCCA	TGGCTTGTTGGTTAACAGC
si‐IGF2BP2#2	ACAGGACTGTCCGTGCTAT	ATAGCACGGACAGTCCTGT
si‐IGF2BP3#1	GCTGGAGCTTCAATTAAGA	TCTTAATTGAAGCTCCAGC
si‐IGF2BP3#2	GCAGGAATTGACGCTGTAT	ATACAGCGTCAATTCCTGC
si‐ACTA1#1	GCCGCGAUCUCACCGACUA TT	GCCGCGAUCUCACCGACUA TT
si‐ACTA1#2	UAGUCGGUGAGAUCGCGGC TT	UAGUCGGUGAGAUCGCGGC TT
si‐ACTA1#3	GGUCGGUAUGGGUCAGAAA TT	GGUCGGUAUGGGUCAGAAA TT
si‐EVL#1	ACGAUGACACCAGUAAGAAAU TT	ACGAUGACACCAGUAAGAAAU TT
si‐EVL#2	AUUUCUUACUGGUGUCAUCGU TT	AUUUCUUACUGGUGUCAUCGU TT
si‐EVL#3	GGACAUCCAGAGAAGACAA TT	GGACAUCCAGAGAAGACAA TT
si‐LSP1#1	CAAGGGCUGAUAACGGCCA TT	CAAGGGCUGAUAACGGCCA TT
si‐LSP1#2	UGGCCGUUAUCAGCCCUUG TT	UGGCCGUUAUCAGCCCUUG TT
si‐LSP1#3	CUAAACCGCUCCAUAGAGA TT	CUAAACCGCUCCAUAGAGA TT
si‐NPY6R#1	UCUUCACCACCUCCCUUUU TT	UCUUCACCACCUCCCUUUU TT
si‐NPY6R#2	AAAAGGGAGGUGGUGAAGA TT	AAAAGGGAGGUGGUGAAGA TT
si‐NPY6R#3	CCUUAAAAUUCAUUAAUUA TT	CCUUAAAAUUCAUUAAUUA TT
si‐PGLYRP2#1	CCGACAUGACGUACGAGAA TT	CCGACAUGACGUACGAGAA TT
si‐PGLYRP2#2	UUCUCGUACGUCAUGUCGG TT	UUCUCGUACGUCAUGUCGG TT
si‐PGLYRP2#3	GCCCCCACAAUCGCCUCUA TT	GCCCCCACAAUCGCCUCUA TT
si‐TCEAL5#1	GAGCUAAGGAAAAAACAGA TT	UCUGUUUUUUCCUUAGCUC TT
si‐TCEAL5#2	GAAUGAAAGAAACCUAGAA TT	UUCUAGGUUUCUUUCAUUC TT
si‐TCEAL5#3	GGGAAGUACAGAAGAUGAA TT	UUCAUCUUCUGUACUUCCC TT
si‐VGF#1	GAGGAGCGAGAGAGUGCAA TT	GAGGAGCGAGAGAGUGCAA TT
si‐VGF#2	UUGCACUCUCUCGCUCCUC TT	UUGCACUCUCUCGCUCCUC TT
si‐VGF#3	CGAGAUUUCAGUCCAAGUA TT	CGAGAUUUCAGUCCAAGUA TT
Negative control	UUCUCCGAACGUGUCACGU TT	ACGUGACACGUUCGGAGAA TT

Abbreviation: qRT‒PCR, quantitative real‐time polymerase chain reaction.

### 2.6 Renal histology and immunohistochemistry

Paraffin‐embedded sections (4 μm) of kidneys were prepared according to the routine protocol. Haematoxylin & eosin (H&E), Masson and Sirius Red staining were performed, and histological examination was performed using a light microscope (Leica) combined with the TissueFAXS panoramic tissue cell quantitative analysis system (TG). Quantitative analysis of Masson staining was performed using ImageJ (NIH). Immunohistochemical staining was conducted following the instructions. Tissue sections were incubated with EVL‐specific antibodies (Proteintech) and goat anti‐rabbit IgG (Horseradish peroxidase‐conjugated AffiniPure) for 12 h (4°C) and 1 h (room temperature), respectively. After diaminobenzidine and haematoxylin staining, the sections were visualised in the same manner.

### 2.7 Immunofluorescence staining and high‐content fluorescence imaging

Paraffin sections (4 μm) or acetone‐fixed cell crawls were treated with antigen repair and incubated with anti‐m6A, anti‐METTL3, anti‐Calbindin, anti‐Aquaporin 3, anti‐Lotus tetragonolobus lectin (LTL), anti‐EVL, anti‐p‐Smad3, anti‐COL1A1 and anti‐α‐SMA antibodies (4°C, overnight). After 40 min of incubation with the appropriate fluorescent secondary antibody at room temperature, 2‐(4‐Amidinophenyl)‐6‐indolecarbamidine dihydrochloride staining was performed. A fluorescence microscope (Olympus IX83) and the TissueFAXS Spectra system were used to acquire fluorescent images. Moreover, HK‐2 cells were uniformly plated in 96‐well plates, induced with TGF‐β1 (5 ng/mL) and treated with 20 screened drug candidates at concentrations of .39, .78 and 1.56 μM. The fluorescence staining procedure was performed as described previously. High‐throughput fluorescence photography was performed by an ImageXpress Micro 4 system (Molecular Devices).

### 2.8 Protein extraction and Western blot analysis

Precooled lysis buffer containing protease inhibitor (APExBIO Technology) was used to prepare kidney tissue or cell lysates. The lysate was subjected to electrophoresis (80 V/30 min followed by 120 V/60 min), transfer (nitrocellulose membrane, 128 V/75 min), blocking (5% skimmed milk, 1.5 h) and incubation (primary antibody, 4°C, overnight; secondary antibody, 2 h, room temperature) (Table S1). Blot signals were recorded by a LiCor/Odyssey system (LI‐COR Biosciences). Quantification of protein blots was performed using ImageJ.

### 2.9 Coimmunoprecipitation

In brief, cells in culture plates were washed with precooled phosphate‐buffered saline solution and lysed by adding 200 μL of 1% NP‐40 lysis buffer/well on ice for 30 min. After collecting the input samples, Smad7 antibody (3.5 μg) and Protein A+G‐Agarose (Bioworld Technology) were incubated overnight at 4°C. The agarose was precipitated, and IgG samples were retained. Subsequently, all samples were subjected to Western blot experiments to explore the binding of EVL and TGF‐βR1 proteins to Smad7.

### 2.10 RNA extraction and quantitative real‐time polymerase chain reaction

TRIzol (Invitrogen) was selected to extract the total cellular or tissue RNA, which was then reverse transcribed to cDNA and detected by real‐time PCR on a CFX96 Real Time RT‒PCR Detection System (Bio‐Rad) using 2X SYBR^®^ Green Pro Taq HS Premix (Accurate Biology). The primers utilised in this study included METTL3, EVL, COL1A1, α‐SMA and GAPDH, and the primer sequences are listed in Table 2. All mRNAs were quantified using GAPDH as an internal control and are shown as the mean ± S.E.M.

### 2.11 Quantification of m6A modification

Total RNA was isolated as described in the RNA extraction procedure, and the quality of RNA processed by deoxyribonuclease I (04716728001, Sigma‒Aldrich) was determined using DeNovix DS‐11 (DeNovix). The level of total m6A in each group was measured using the EpiQuik m6A RNA Methylation Quantification Kit (P‐9005, EpigenTek) according to the manufacturer's protocol. Briefly, 200 ng of RNA was added dropwise to the assay wells. Afterwards, appropriate concentrations of capture and detection antibodies were added to each well. The SpectraMax iD3 multifunctional enzyme analyser (Molecular Devices) was used to detect the absorbance of the m6A quantitation at 450 nm. A standard curve was plotted to calculate the concentration.

### 2.12 Dot blot

The abundance of m6A in the poly‐A tail of total RNA was detected using an RNA m6A dot blot assay. In brief, total RNA in tissues or cells was extracted on ice and denatured at 70°C for 5 min. The prepared RNA (200 and 400 ng, respectively) was added dropwise onto a nylon membrane (FFN10, Beyotime) with duplicate copies. The membranes were cross‐linked by ultraviolet exposure for 1 h, blocked with 5% nonfat milk and then incubated with m6A antibody (68055‐1‐Ig, Proteintech) overnight. Western blot analysis was performed to obtain dot blot images. In parallel, methylene blue staining was utilised as a loading control.

### 2.13 RNA immunoprecipitation

The RNA immunoprecipitation (RIP) study was conducted with the BersinBio^™^ RNA RIP kit (Bes5101) following the instructions. Briefly, a sufficient number of HK‐2 cells (2 × 10^7^/sample) were collected and lysed on ice with polysome lysis buffer (containing protease inhibitor and RNase inhibitor), and DNA was removed by adding DNase salt stock. The prepared cell lysates were incubated with anti‐IGF2BP2 antibody (5 μg) or IgG (1 μg/μL) for 16 h at 4°C and then with 20 μL of Protein A/G magnetic beads for 1 h at 4°C. The A/G beads containing RNA‒protein complexes were then eluted with 200 μL of polysome elution buffer containing proteinase K at 55°C for 1 h. RNA concentrations were extracted and verified. Quantitative real‐time polymerase chain reaction (qRT‒PCR) analysis of IGF2BP2 antibody‐enriched RNA was calculated by normalisation of the input.

### 2.14 m6A‐MeRIP‐Seq

m6A‐MeRIP‐Seq was performed according to the mouse m6A–mRNA epitranscriptome microarray protocol. Briefly, total RNA (1–10 μg) was extracted, purified and quantified, and its integrity was determined with a Bioanalyser 2100 (Agilent). An anti‐m6A antibody (202003, Synaptic Systems) was used for the RIP of total RNA. The immunoprecipitated magnetic beads (11203D, Invitrogen) with RNA enriched in m6A antibody were eluted as ‘(IP: elute)’. The unenriched RNA in the supernatant was extracted as ‘IP: Supernatant’. RNAs from ‘IP: Supernatant’ and ‘IP: elute’ were labelled with Cy3 and Cy5 as separately reacted cRNAs, respectively. These cRNAs were hybridised using an Arraystar mRNA epitranscriptomic microarray (8×60K, Arraystar). An Agilent scanner (G2505C) and feature extraction software were used to scan and analyse the hybridisation arrays in a two‐colour channel format. MeRIP and RNA epitranscriptome microarray analysis were provided by Aksomics.

### 2.15 MeRIP‐qPCR

The MeRIP was implemented as previously mentioned. Briefly, the polymorph‐purified RNA was denatured at 65°C for 10 min and immediately transferred to ice. Subsequent reactions were performed in a 300 μL IP buffer system (27 μL of RNA sample, 3 μL of RNase inhibitor [Y9240L, Enzymatics], 60 μL of 5× IP buffer [750 mM NaCl, 50 mM Tris–HCl and .5% NP‐40], 2 μL of anti‐m6A antibody [Synaptic Systems, 202003] and 210 μL of H_2_O) (4°C, 2 h). Then, 20 μL of Dynabeads^™^ M‐280 (11203D, Invitrogen) was mixed thoroughly with the RNA–antibody mixture and incubated for 2 h at 4°C with rotation. The RNA enriched on the beads was eluted using 200 μL of elution buffer (50°C, 1 h). Afterwards, RNA was obtained from the supernatant and IP samples using the equivalent volume of phenol chloroform (isoamyl alcohol:chloroform:phenol = 1:24:25), and the methylated mRNA was precipitated by precipitation (1/10 volume of 3 M sodium acetate, 2.5 volumes of 100% ethanol and 5 μg glycogen, −80°C, overnight). The m6A enrichment was quantified by qRT‒qPCR and was calculated by normalisation of the input.

### 2.16 RNA stability

Actinomycin D (5 μg/mL) was added to the cultured cells to terminate mRNA transcription for the stability assay. After 0, 1, 3 and 6 h of actinomycin D treatment, the cells were collected, and residual levels of EVL were measured by qRT‒PCR.

### 2.17 Virtual screening and molecular docking

The crystal structure of the human METTL3–METTL14 complex (PDB ID: 6TTW) was accessed from the Protein Data Bank. The commercial database (L6810) containing 2688 TCM monomers was provided by TargetMol^®^ (Topscience). Molecular docking models were constructed using Schrodinger v11.5 software (Schrödinger, Inc.) to molecularly dock the compounds (Libdock and CDOCKER). The docking protocol was validated by the eutectic ligand S‐adenosylmethionine competitive small molecule inhibitor STM2457 with the catalytic structural domain of the complex. All compounds in the library were ranked by scoring metrics, namely, the docking score and glide score. The top 20 candidate compounds (docking scores ranging from 7.488/7.488 to 5.511/5.513) were considered for further cytotoxicity and activity testing, and they were screened for the best TCM monomer.

### 2.18 Cellular thermal shift assay

Cellular thermal shift assay (CETSA) trials were conducted following the established protocol. Briefly, cells were harvested and lysed with RIPA lysis buffer on ice after 36 h of treatment with or without isoforsythiaside. Total protein in each group was quantified with a BCA protein concentration assay kit and equally distributed into different PCR tubes by adding protein sample loading buffer (Beyotime). The samples were denatured by heating for 8 min at selected temperatures (RT, 42°C, 47°C, 52°C, 57°C and 62°C) and placed in liquid nitrogen for three cycles of repeated freeze‒thawing. The samples were centrifuged, and the supernatant was analysed by Western blotting.

### 2.19 Statistical analysis

Data normalisation was performed as follows: the measured values were divided by the corresponding internal control values to arrive at a new value. Then, the mean of the control or sham group was calculated. Finally, the normalised data were obtained by dividing the new values of each group by the mean value. Graphs were created utilizing GraphPad Prism (v8.3.0; GraphPad Software). Data were statistically analysed using SPSS Statistics (v26.0; IBM). The data were subjected to the Shapiro‒Wilk method for testing normal distribution, and quantitative data of normal distribution are reported as the mean ± S.E.M. Independent samples *t* tests, one‐way analysis of variance (ANOVA) as well as Tukey's post hoc test were employed to evaluate differences in means across different groups. A *p*‐value <.05 was deemed statistically significant.

## 3 RESULTS

### 3.1 METTL3 and m6A modification are highly induced in human nephrectomised tissues, mouse models and renal tubular epithelial cells in response to renal fibrosis

**FIGURE 1:**
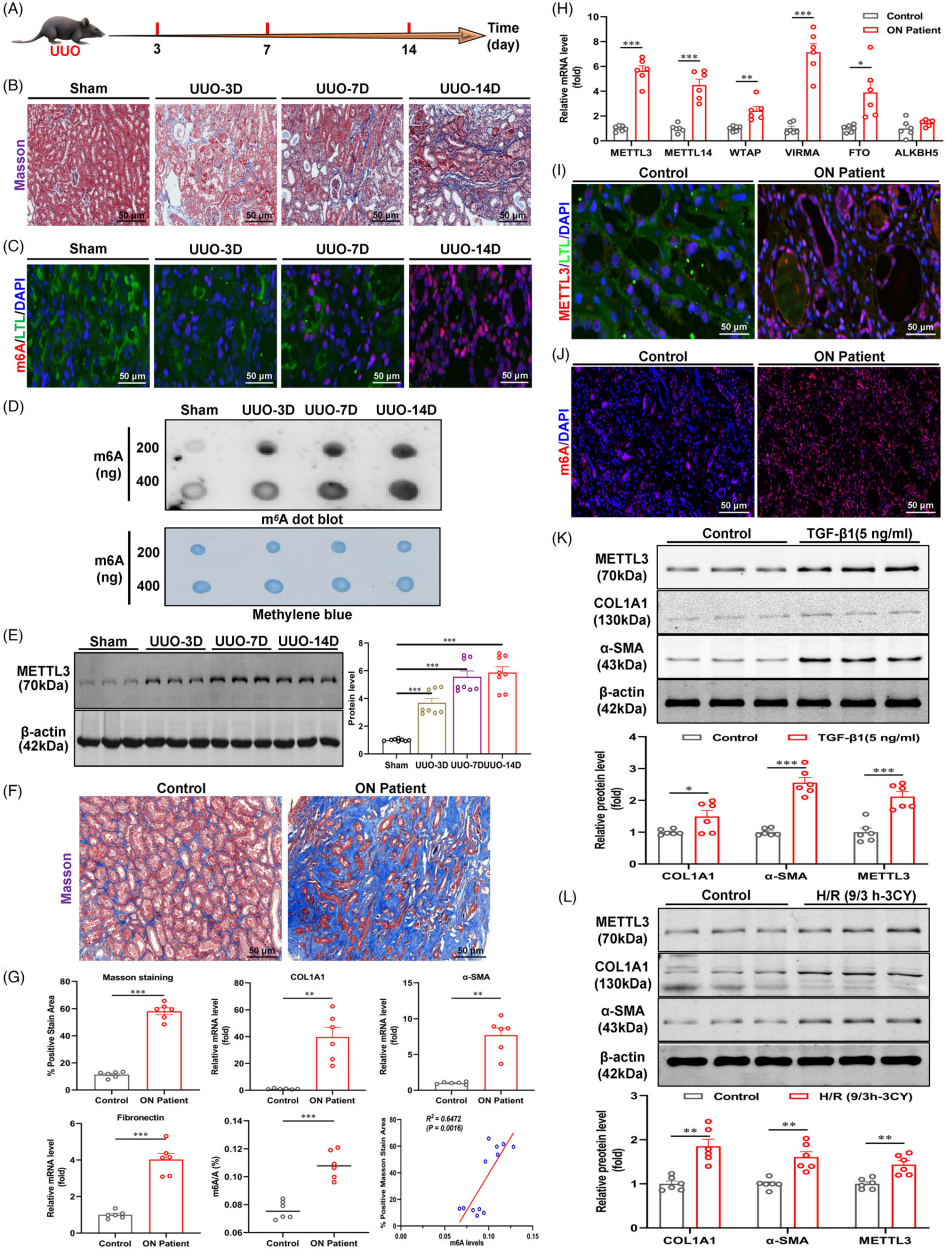
Levels of methyltransferase‐like 3 (METTL3) and *N* ^6^‐methyladenosine (m6A) modifications increase in human renal tissues, mouse models and renal tubular epithelial cells in response to profibrotic stimuli. (A) Schematic diagram of the unilateral ureteral obstruction (UUO)‐induced mouse model of renal fibrosis. (B) Representative Masson staining of renal tissue from UUO mice (*n* = 6). Scale bar = 50 μm. (C) Representative immunofluorescence staining of m6A and Lotus tetragonolobus lectin (LTL) in renal tissues of UUO mice (*n* = 3). LTL was used to label the proximal tubule. Scale bar = 50 μm. (D) Dot blot assay showed that UUO treatment increases m6A abundance in renal tissues. (E) Expression of METTL3 protein in renal tissue of UUO mice (*n* = 8). (F) Representative Masson staining of renal tissue from obstructive nephropathy (ON) patients (*n* = 6). Scale bar = 50 μm. (G) Masson staining score, mRNA levels of fibrotic indicators and total m6A levels in renal tissues of ON patients (*n* = 6). (H) mRNA levels of multiple m6A regulators in renal tissues of ON patients (*n* = 6). (I) Representative immunofluorescence staining of METTL3 and LTL in renal tissues of ON patients. LTL was used to label the proximal tubules (*n* = 6). Scale bar = 50 μm. (J) Representative immunofluorescence staining of m6A modification in renal tissues of ON patients (*n* = 6). Scale bar = 50 μm. (K) Protein levels of METTL3 and fibrotic indicators in transforming growth factor‐β1 (TGF‐β1)‐treated HK‐2 cells (*n* = 6). (L) Protein levels of METTL3 and fibrotic indicators in hypoxia–reoxygenation (H/R)‐treated HK‐2 cells (*n* = 6). Sham represents mice subjected to sham operation. Control represents renal cell carcinoma paracellular tissue or untreated HK‐2 cells. Data represent the mean ± S.E.M. of at least six independent experiments in vitro and six to eight mice in vivo. Statistically significant differences were determined by independent sample *t* test and one‐way analysis of variance (ANOVA) followed by Tukey's post hoc test. ^*^ *p* < .05, ^**^ *p* < .01 and ^***^ *p* < .001.

The model establishment schedules are shown in Figures 1A and S1A. H&E staining showed significant fibrotic features, such as inflammatory cell infiltration, luminal hyaline tubular pattern and loss of some tubular structures in the kidney tissues of UUO and I/R mice models, which gradually increased with disease progression (Figure S1B,F). Masson and Sirius Red staining revealed progressively increased deposition of collagen fibres in interstitial areas of the kidney (Figures 1B and S1B,F). In addition, remarkably elevated m6A modifications in fibrotic kidney tissues were confirmed by immunofluorescence staining and dot blot assay (Figures 1C,D and S1C,D), which was consistent with immunofluorescence staining for METTL3 (Figure S3A,B). Moreover, the protein levels of METTL3 were upregulated in the UUO and I/R‐induced mouse models compared to the sham group, which was aligned with the trend of fibrotic indicators (COL1A1 and α‐SMA) (Figures 1E and S1E,G). In both mouse models, there were no alterations in the protein levels of methyltransferases and demethylases beyond METTL3 that were consistent with m6A modifications. Importantly, METTL3 was highly induced in the fibrotic kidney tissues of ON patients and was predominantly localised in the renal tubular area, which was consistent not only with the changes in fibrotic indicators, Masson and Sirius Red staining, but also with the alterations in total m6A modifications (Figures 1F–J and S2A). Correlation analysis further revealed that both m6A modifications and METTL3 expression levels in kidney tissues of ON patients were positively correlated with fibrosis indicators and Masson staining (Figures 1G and S2B–D). Similar results were obtained in TGF‐β1‐ and H/R‐stimulated HK‐2 cells. Aligned with the fibrotic indicators being highly induced, METTL3 and m6A modifications were also induced and highly expressed in HK‐2 cells by different stimuli, including H/R and TGF‐β1 (Figures 1K,L and S3C–F). Furthermore, immunofluorescence staining of METTL3 in different segments of renal tubules from kidney tissues of UUO‐ and I/R‐induced fibrotic mice showed that METTL3 was not only hyperexpressed in the proximal tubule region, but also significantly elevated in the collecting duct and distal duct compared with kidney tissues of the sham group (Figure S3G–I).

### 3.2 METTL3 deletion alleviates UUO‐ and I/R‐induced renal fibrosis in mice

**FIGURE 2:**
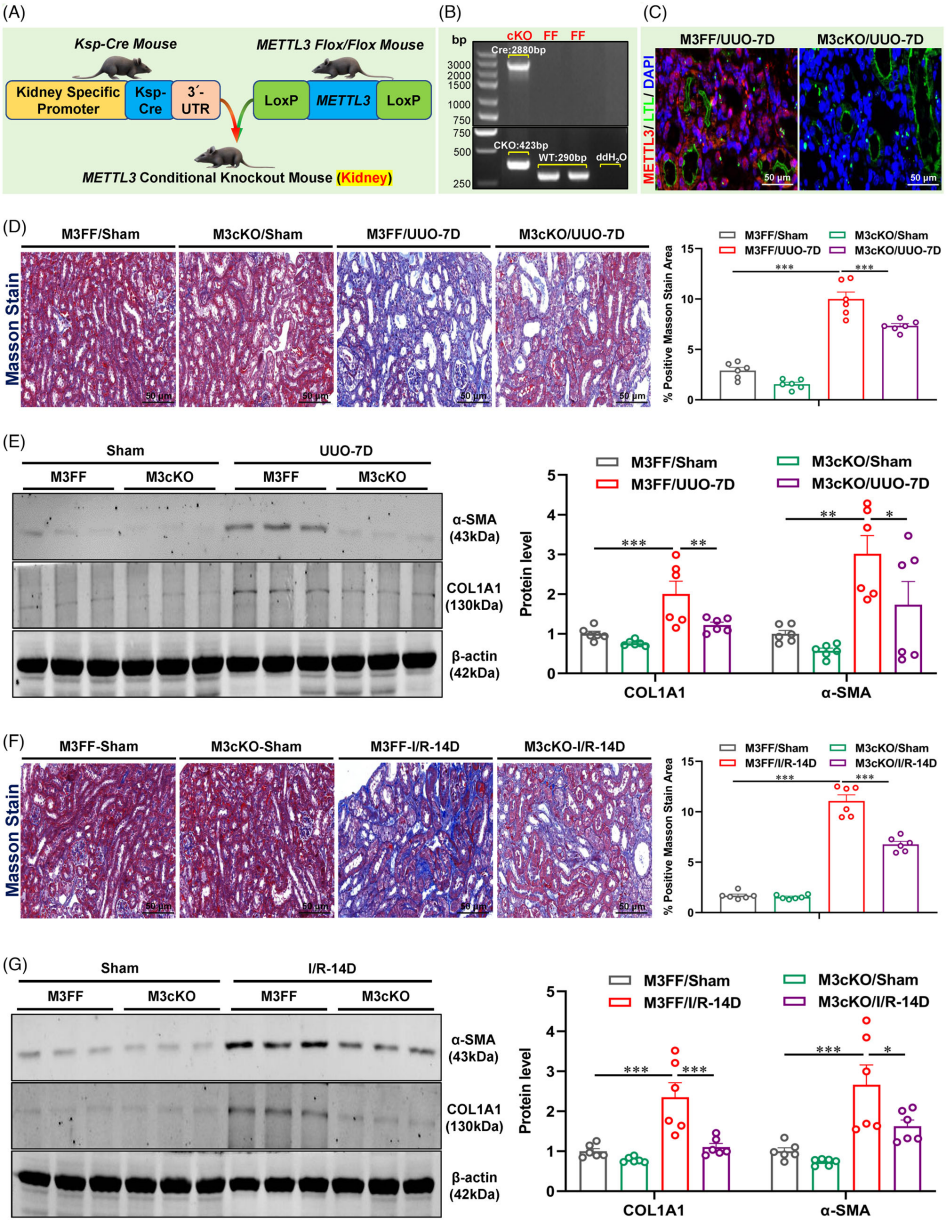
Methyltransferase‐like 3 (METTL3) deletion alleviates unilateral ureteral obstruction (UUO)‐ and ischemia‒reperfusion (I/R)‐induced renal fibrosis in mice. (A) Schematic illustrating the genetic approach used for generating METTL3 conditional knockout (cKO) mice. (B) METTL3 cKO was confirmed by agarose gel electrophoresis detection of genomic DNA. (C) Representative immunofluorescence staining of METTL3 and Lotus tetragonolobus lectin (LTL) in METTL3*^Flox/Flox^* and METTL3 cKO mice with UUO. LTL was used to label the proximal tubules (*n* = 6). Scale bar = 50 μm. (D) Representative Masson staining to assess the effect of METTL3 cKO on renal pathology and fibrosis in UUO mice (*n* = 6). Scale bar = 50 μm. (E) Effect of METTL3 cKO on the protein levels of renal fibrosis indicators in UUO mice (*n* = 6). (F) Representative Masson staining to assess the effect of METTL3 cKO on renal pathology and fibrosis in I/R mice (*n* = 6). Scale bar = 50 μm. (G) Effect of METTL3 cKO on the protein levels of renal fibrosis indicators in I/R mice (*n* = 6). Sham represents mice subjected to sham operation. Data represent the mean ± S.E.M. of at least six to eight mice in vivo. Statistically significant differences were determined by independent sample *t* test and one‐way analysis of variance (ANOVA) followed by Tukey's post hoc test. ^*^ *p* < .05, ^**^ *p* < .01 and ^***^ *p* < .001.

To confirm the functions of METTL3 in vivo, we used the *Cre‐LoxP* recombinant system based on the C57BL/6 background to construct *Ksp‐Cre* METTL3*^fl/fl^* mice by mating kidney‐specific promoter (Cadherin‐16)‐driven *Cre* (*Ksp‐Cre*) mice with METTL3*^fl/fl^* mice, resulting in METTL3 cKO mice (Figure 2A). All of the subsequent experiments involving METTL3 cKO mice were performed using PCR for tail genotyping (Figure 2B). The dual immunofluorescence staining of LTL with METTL3 also verified that METTL3 was deleted from tubular epithelial cells (TECs) in the kidney (Figure 2C). Masson staining and quantitative analysis demonstrated that deficiency of METTL3 significantly diminished UUO‐induced collagen fibre deposition in the renal interstitium (Figure 2D). Western blot assays exhibited that the protein levels of COL1A1 and α‐SMA were markedly raised in UUO‐induced fibrotic kidney tissues, while METTL3 cKO significantly decreased the protein levels of these fibrosis indicators (Figure 2E). These results have been further validated in a mouse renal fibrosis model induced by I/R (Figure 2F,G). Together, these results suggest that METTL3 is a key mediator of renal fibrosis.

### 3.3 Silencing METTL3 reduces fibrotic responses in TGF‐β1‐ and H/R‐treated HK‐2 cells

**FIGURE 3:**
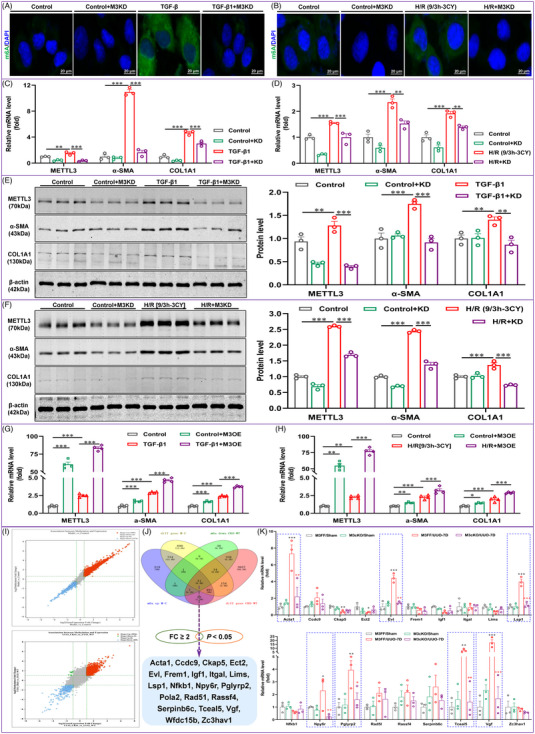
Anti‐renal fibrosis effects of methyltransferase‐like 3 (METTL3) and screening of potential *N* ^6^‐methyladenosine (m6A)‐modified target genes. (A and B) METTL3 knockdown reduced m6A modifications in transforming growth factor‐β1 (TGF‐β1)‐ and hypoxia–reoxygenation (H/R)‐treated HK‐2 cells (*n* = 3). (C and D) METTL3 knockdown significantly attenuated the mRNA levels of fibrotic indicators in TGF‐β1‐ and H/R‐treated HK‐2 cells (*n* = 3). (E and F) METTL3 knockdown significantly attenuated the protein expression of fibrotic indicators in TGF‐β1‐ and H/R‐treated HK‐2 cells (*n* = 3). (G and H) Forced expression of METTL3 significantly exacerbated the fibrotic responses of TGF‐β1 and H/R‐treated HK‐2 cells (*n* = 4). (I) Correlation analysis of differentially altered mRNAs with mRNA m6A modifications. (J) Filtering of the differential genes by fold change ≥2 and *p* < .05 criteria as well as filtering of potential target genes for METTL3‐mediated m6A modifications by Venn diagram. (K) Validation of the mRNA expression of potential target genes in the fibrotic kidney tissue of METTL3 conditional knockout (cKO) unilateral ureteral obstruction (UUO) mice (*n* = 3). Control represents untreated HK‐2 cells. Data represent the mean ± S.E.M. of at least three independent experiments in vitro. Statistically significant differences were determined by independent sample *t* test and one‐way analysis of variance (ANOVA) followed by Tukey's post hoc test. ^*^ *p* < .05, ^**^ *p* < .01 and ^***^ *p* < .001.

After clarifying the importance of METTL3 in the mouse renal fibrosis models induced by UUO and I/R, we also assessed the function of METTL3 in TGF‐β1 (5 ng/mL)‐ and H/R (9/3 h, three cycles)‐induced fibrotic responses in HK‐2 cells. As presented in Figure 3A,B, m6A modification was markedly enhanced in HK‐2 cells after treatment with TGF‐β1 and H/R compared to the control, whereas METTL3 silencing significantly attenuated the abnormally elevated level of m6A modification. Moreover, we found that the significantly enhanced m6A modification in HK‐2 cells induced by TGF‐β1 with H/R was more enriched in the cytoplasm than in the nucleus. Following validation of the silencing effect of METTL3 siRNA in HK‐2 cells, Western blot and qRT‒PCR analyses demonstrated that TGF‐β1 and H/R induced a significant increase in both the mRNA and protein levels of fibrotic indicators, such as α‐SMA and COL1A1, suggesting the development of a fibrotic response in HK‐2 cells. Moreover, METTL3 silencing significantly reduced the elevated mRNA and protein expression levels of fibrosis indicators caused by both stimuli, whereas forced expression of METTL3 exacerbated the fibrotic responses (Figure 3C–H). Collectively, METTL3 is a crucial mediator of the fibrotic response of HK‐2 cells induced by different profibrotic stimuli.

### 3.4 Identification of EVL as a direct target of METTL3‐mediated m6A modification during renal fibrosis

**FIGURE 4:**
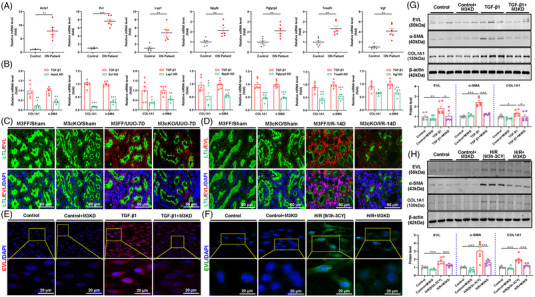
Ena/VASP‐like (EVL) is a potential target gene for methyltransferase‐like 3 (METTL3)‐mediated *N* ^6^‐methyladenosine (m6A) modification in renal fibrosis. (A) Validation of mRNA expression for potential target genes in kidney tissues from obstructive nephropathy (ON) patients (*n* = 6). (B) Effect of potential target gene silencing on the mRNA levels of fibrosis indicators in transforming growth factor‐β1 (TGF‐β1)‐treated HK‐2 cells (*n* = 6). (C) Representative immunofluorescence staining of EVL and Lotus tetragonolobus lectin (LTL) in kidney tissues of unilateral ureteral obstruction (UUO) mice with METTL3 conditional knockout (cKO). LTL was used to label the proximal tubules (*n* = 3). Scale bar = 50 μm. (D) Representative immunofluorescence staining of EVL and LTL in kidney tissues of ischemia‒reperfusion (I/R) mice with METTL3 cKO. LTL was used to label the proximal tubules (*n* = 3). Scale bar = 50 μm. (E and F) Representative immunofluorescence staining to observe the effect of METTL3 knockdown on EVL expression in TGF‐β1‐ and hypoxia–reoxygenation (H/R)‐treated HK‐2 cells (*n* = 3). Scale bar = 20 μm. (G and H) Western blot analyses of the effect of METTL3 knockdown on EVL and fibrotic indicators in TGF‐β1‐ and H/R‐treated HK‐2 cells (*n* = 6). Sham represents mice subjected to sham operation. Control represents renal cell carcinoma paracellular tissue or untreated HK‐2 cells. Data represent the mean ± S.E.M. of at least three to four independent trials. Statistically significant differences were determined by independent samples *t* test, one‐way analysis of variance (ANOVA) and Tukey's post hoc test. ^*^ *p* < .05, ^**^ *p* < .01 and ^***^ *p* < .001.

To explore the specific characteristics of m6A modifications that regulate mRNA in renal fibrosis and identify potential targets for METTL3, we performed m6A‐Seq and RNA‐Seq on the kidneys of UUO mice. In total, 4116 and 3086 genes were upregulated and downregulated, respectively, in UUO‐induced fibrotic kidney tissues compared to normal kidney tissues (Figure S3J,K). Correlation analysis of mRNA and m6A modifications showed that 3909 genes were significantly upregulated with correlated m6A modification levels, while 2978 genes were significantly downregulated with correlated m6A modification levels, and there was no reverse change in gene expression and m6A modification (Figure 3I). Compared to UUO‐induced fibrotic kidney tissues in wild‐type mice, a total of 3262 genes showed increased expression levels, and 1276 genes showed decreased expression levels in renal tissues after METTL3 cKO (Figure S3J–K). Correlation analysis of mRNA and m6A modifications showed that 3094 genes and 409 genes were significantly upregulated and downregulated with correlated m6A modification levels, respectively, and only nine genes with opposite expression and m6A modification levels existed (Figure 3I). The aforementioned findings implied that the changes in m6A modification positively regulated gene expression. Importantly, we performed intersection analysis of the two sequencing results with a fold change (FC) ≥2 and a *p*‐value <.05 as the filtering criteria using Venn diagrams. After excluding logical faults, we screened a total of 19 potential genes (Figure 3J). Validation of METTL3 cKO kidney tissues using qRT‒PCR revealed seven genes, including ACTA1, EVL, LSP1, NPY6R, PGLYRP2, TCEAL5 and VGF, which showed consistent expression changes in each group with the sequencing results (Figure 3K). qRT‒PCR also validated the above seven potential target genes in human fibrotic kidney tissue (Figure 4A).

**FIGURE 5:**
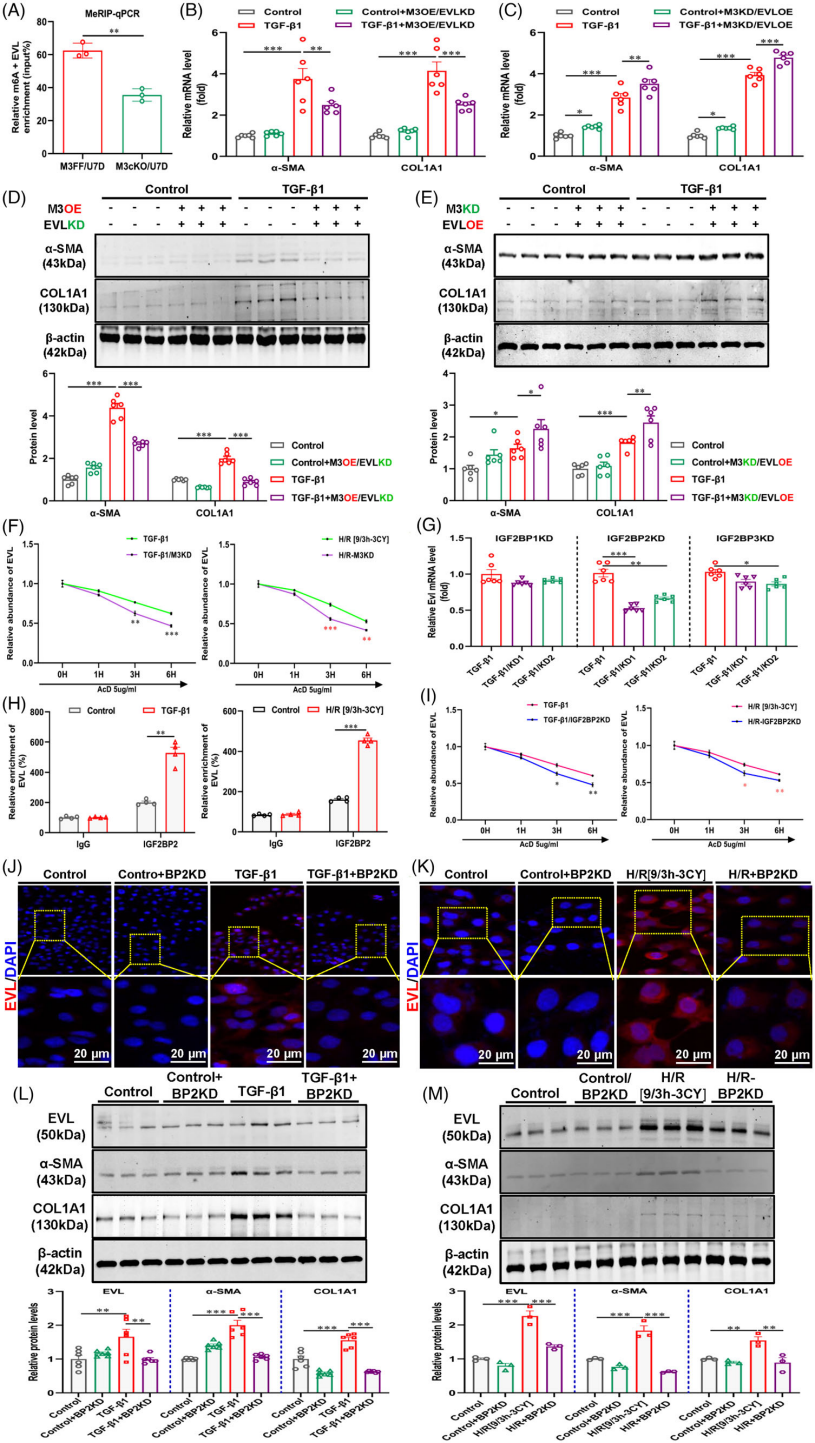
Methyltransferase‐like 3 (METTL3) directly enhances the *N* ^6^‐methyladenosine (m6A) modification and stability of Ena/VASP‐like (EVL) mRNA in an insulin‐like growth factor 2 mRNA‐binding protein 2 (IGF2BP2)‐dependent manner. (A) Methylation RNA immunoprecipitation (MeRIP)‒qPCR analyses of the altered m6A levels of the EVL gene in different groups (*n* = 3). (B) Quantitative real‐time polymerase chain reaction (qRT‒PCR) analyses of the effects of simultaneous METTL3 overexpression and EVL knockdown on the fibrotic response of HK‐2 cells (*n* = 6). (C) qRT‒PCR analyses of the effects of simultaneous METTL3 knockdown and EVL overexpression on the fibrotic response of HK‐2 cells (*n* = 6). (D) Rescue of METTL3 expression in EVL knockdown HK‐2 cells protected against transforming growth factor‐β1 (TGF‐β1)‐induced fibrotic response (*n* = 6). (E) Rescue of EVL expression in METTL3 knockdown HK‐2 cells restored fibrotic response (*n* = 6). (F) qRT‒PCR analyses of the decay rate of EVL mRNA after actinomycin D (5 μg/mL) treatment in METTL3 knockdown HK‐2 cells. (G) qRT‒PCR analysis of the effect of IGF2BP silencing on EVL mRNA levels in TGF‐β1‐treated HK‐2 cells (*n* = 6). (H) RNA immunoprecipitation (RIP) assays showing the direct binding between the IGF2BP2 protein and EVL mRNA in TGF‐β1‐ and hypoxia–reoxygenation (H/R)‐treated HK‐2 cells (*n* = 4). (I) qRT‒PCR analyses of the decay rate of EVL mRNA after actinomycin D (5 μg/mL) treatment in IGF2BP2 knockdown HK‐2 cells. (J and K) Representative immunofluorescence staining analyses of EVL expression after IGF2BP2 knockdown in TGF‐β1‐ and H/R‐treated HK‐2 cells (*n* = 3). Scale bar = 20 μm. (L and M) Western blot analyses of the protein levels of EVL and fibrotic indicators after IGF2BP2 knockdown in TGF‐β1‐ and H/R‐treated HK‐2 cells (*n* = 6/3). Control represents untreated HK‐2 cells. Data represent the mean ± S.E.M. of at least three to four independent trials. Statistically significant differences were determined by independent samples *t* test, one‐way analysis of variance (ANOVA) and Tukey's post hoc test. ^*^ *p* < .05, ^**^ *p* < .01 and ^***^ *p* < .001.

The seven potential genes were selected to explore their potential functions. Initially, we screened the appropriate siRNAs for these potential genes by qRT‒PCR (Figure S4A). We then silenced these potential genes in TGF‐β1‐treated HK‐2 cells and found that EVL silencing reduced the fibrotic response more significantly than ACTA1, LSP1, NPY6R, PGLYRP2, TCEAL5 and VGF silencing (Figure 4B). Hence, EVL was considered a potential target gene for METTL3‐mediated m6A modification. Bioinformatics analysis revealed the presence of multiple m6A modification sites on EVL mRNA, which confirmed the possibility of m6A modifications on EVL mRNA (Figure S4B). Through MeRIP‐qPCR, we verified that the m6A antibody was enriched in EVL mRNA (Figure 5A). Moreover, both Western blot and qRT‒PCR experiments revealed that rescuing METTL3 expression in EVL‐knockdown HK‐2 cells did not exacerbate the TGF‐β1‐induced fibrotic response (Figure 5B,D). In contrast, rescuing EVL expression in METTL3 knockdown HK‐2 cells restored or even increased the fibrotic response (Figure 5C,E). These rescue experiments indicated that EVL is a direct target gene of METTL3‐mediated m6A modification. Additionally, immunofluorescence staining and Western blot assays revealed that EVL was markedly upregulated in human fibrotic kidney tissue and enriched mainly in the tubular region of ON patients (Figure S4C,D) and mouse fibrotic kidneys, which was consistent with the METTL3‐mediated m6A modification profile (Figure S4E,F). METTL3 cKO significantly reduced EVL upregulation in fibrotic kidney tissues (Figure 4C,D). In line with the in vivo experiments, METTL3 silencing also significantly downregulated EVL expression and attenuated the fibrotic response of HK‐2 cells induced by TGF‐β1 and H/R (Figure 4E–H). Collectively, these results suggest that EVL is an important direct target gene for METTL3‐mediated m6A modifications during renal fibrosis.

### 3.5 METTL3 enhances the m6A modification and stability of EVL mRNA in an IGF2BP2‐dependent manner

In TGF‐β1‐ and H/R‐stimulated HK‐2 cells, METTL3 deficiency significantly diminished the degradation half‐life of EVL mRNA, indicating that METTL3‐mediated m6A modification promoted the stability of EVL mRNA (Figure 5F). Considering the role played by reading proteins in the process of m6A modification and the nonnegligible role of IGF2BP family members in regulating the translation and stability of m6A‐modified mRNAs,^26^ we examined the involvement of IGF2BP1, IGF2BP2 and IGF2BP3 in EVL mRNA stability. For IGF2BP1, IGF2BP2 and IGF2BP3, we designed two siRNAs and confirmed the interference efficiency of these siRNAs by qRT‒PCR (Figure S5B). Moreover, silencing IGF2BP2, but not IGF2BP1 or IGF2BP3, significantly inhibited the mRNA expression of EVL (Figure 5G). RIP assay performed with an anti‐IGF2BP2 antibody further validated the interaction between IGF2BP2 and EVL mRNA in TGF‐β1 and H/R‐treated HK‐2 cells (Figure 5H). RNA stability assays showed that silencing IGF2BP2 followed by treatment with actinomycin D (5 μg/mL) significantly reduced the degradation half‐life of EVL mRNA in HK‐2 cells treated with TGF‐β1 and H/R (Figure 5I), suggesting that IGF2BP2 plays a pivotal role in maintaining the stability of EVL mRNA. Immunofluorescence staining combined with Western blot analysis showed that silencing IGF2BP2 not only significantly decreased the protein expression of fibrosis indicators, such as α‐SMA and COL1A1, in HK‐2 cells treated with TGF‐β1 and H/R, but also remarkably inhibited the level of EVL protein (Figure 5J–M). These results suggested that METTL3‐dominated EVL m6A modification enhances its mRNA stability and protein expression, in which IGF2BP2 exerts an indispensable auxiliary role, and these actions significantly promote the fibrotic response of HK‐2 cells.

### 3.6 Blocking EVL attenuates the fibrotic response of TGF‐β1‐ and H/R‐treated HK‐2 cells

**FIGURE 6:**
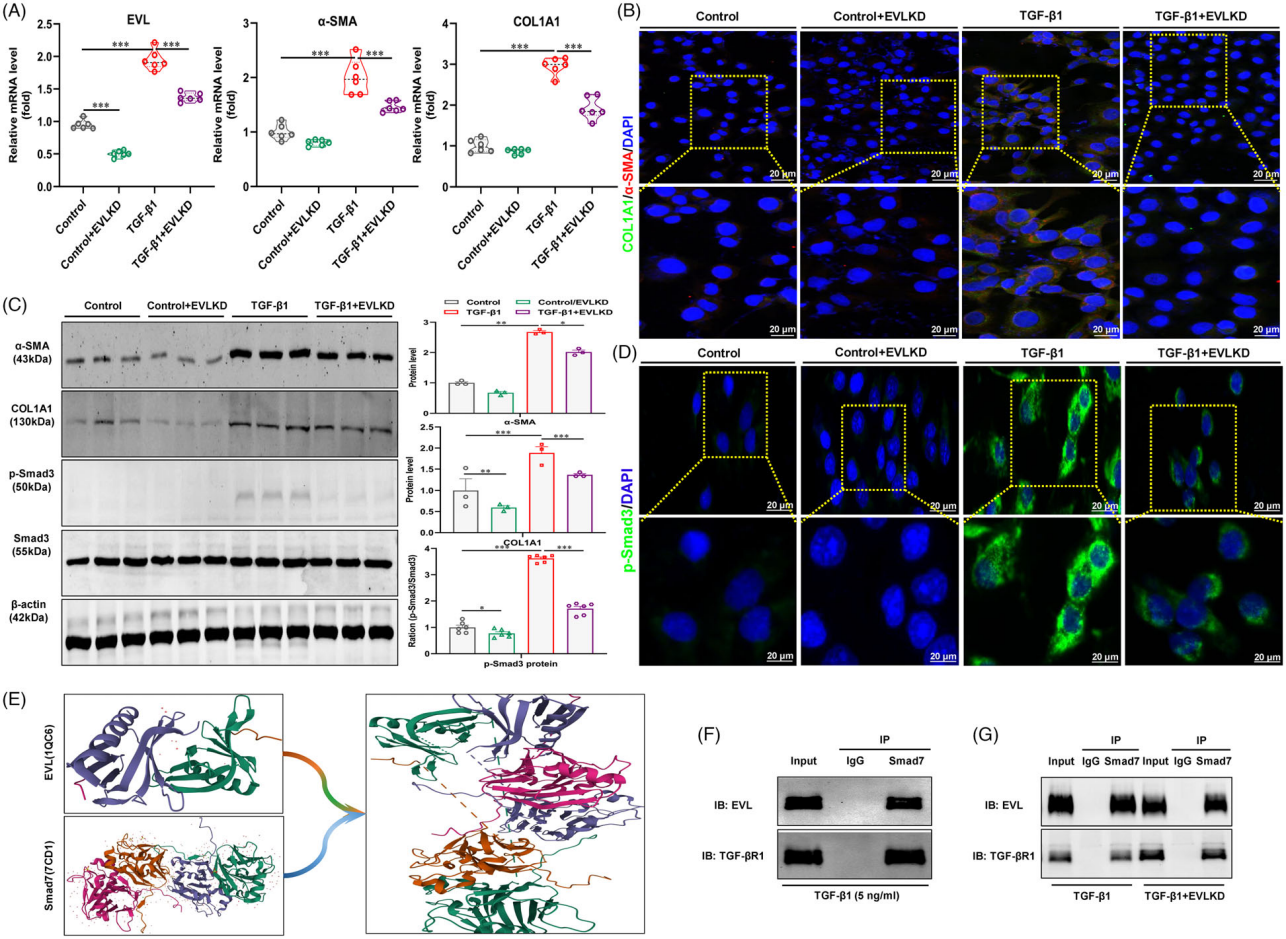
Silencing of Ena/VASP‐like (EVL) attenuates the fibrotic response of transforming growth factor‐β1 (TGF‐β1)‐treated HK‐2 cells probably through a Smad7/TGF‐β1/Smad3 mechanism. (A) Quantitative real‐time polymerase chain reaction (qRT‒PCR) analyses of fibrotic indicators in TGF‐β1‐treated HK‐2 cells with EVL knockdown (*n* = 6). (B) Representative immunofluorescence staining analyses of fibrotic indicators in TGF‐β1‐treated HK‐2 cells with EVL knockdown (*n* = 3). Scale bar = 20 μm. (C) Western blot analyses of the fibrotic indicators, p‐Smad3 and Smad3 in TGF‐β1‐treated HK‐2 cells with EVL knockdown (*n* = 3/6). (D) Representative immunofluorescence staining analyses of p‐Smad3 in TGF‐β1‐treated HK‐2 cells with EVL knockdown (*n* = 3). Scale bar = 20 μm. (E) Molecular docking of the EVL protein to the Smad7 protein. (F) Coimmunoprecipitation (co‐IP) assay detected the protein interactions of EVL, TGF‐βR1 and Smad7 in TGF‐β1‐treated HK‐2 cells. (F) co‐IP assay assessed the effect of EVL silencing on the protein interactions of EVL, TGF‐βR1 and Smad7 in TGF‐β1‐treated HK‐2 cells. Control represents untreated HK‐2 cells. Data represent the mean ± S.E.M. of at least three to four independent experiments. Statistically significant differences were determined by independent sample *t* test and one‐way analysis of variance (ANOVA) followed by Tukey's post hoc test. ^*^ *p* < .05, ^**^ *p* < .01 and ^***^ *p* < .001.

After defining the role and mechanism of METTL3‐dominated m6A modification of EVL mRNA in renal fibrosis, we investigated the function and possible mechanism of EVL. Initially, we established EVL knockdown HK‐2 cells and verified their effectiveness by qRT‒PCR (Figures 6A and S5C). In TGF‐β1‐treated HK‐2 cells, we found upregulated EVL at both the mRNA and protein levels, whereas knockdown of EVL not only significantly reduced the mRNA expression of fibrotic indicators, such as COL1A1 and α‐SMA, but also reduced their protein expression levels (Figure 6A–C). Similar results were confirmed in H/R‐stimulated HK‐2 cells (Figure S5C–E). These findings suggested that upregulated EVL may promote the expression of fibrosis‐related proteins with the progression of renal fibrosis but that knockdown of EVL significantly alleviates the fibrotic response in HK‐2 cells.

Upon identification of the role of EVL in renal fibrosis, we further investigated the possible mechanism. Western blot analysis showed that EVL silencing reduced the expression of fibrosis‐associated proteins coupled with a significant decrease in p‐Smad3 protein expression, but Smad3 expression showed only slight changes, which directly led to a decline in the p‐Smad3/Smad3 ratio compared to that in TGF‐β1‐treated HK‐2 cells (Figure 6C). Similar results were validated in H/R‐treated HK‐2 cells (Figure S5D). Immunofluorescence staining revealed that TGF‐β1 and H/R treatment significantly upregulated the expression level of p‐Smad3, which was accompanied by a marked increase in its nuclear translocation, while knockdown of EVL effectively diminished the expression of p‐Smad3 protein and decreased its nuclear translocation (Figures 6D and S5F). These findings implied that EVL may promote the fibrotic response by modulating TGF‐β1/Smad3 signalling. We then predicted a potential interaction between EVL and Smad7 protein by molecular docking experiments (Figure 6E). Meanwhile, coimmunoprecipitation (co‐IP) assays showed that Smad7 could not only bind to the EVL protein but also to the TGF‐βR1 protein in TGF‐β1‐treated HK‐2 cells (Figure 6F). Importantly, a co‐IP assay performed after silencing EVL exhibited decreased binding of EVL to the Smad7 protein and increased binding of Smad7 to the TGF‐βR1 protein (Figure 6G).

Mechanistically, given the negative regulatory role of Smad7 in TGF‐β1/Smad signalling, we speculated that upregulated EVL can bind to Smad7 in HK‐2 cells treated with TGF‐β1 and H/R, which may reduce the binding of Smad7 to the TGF‐βR1 protein to decrease the inhibitory effect of Smad7 on TGF‐β1/Smad signalling. These changes increase both the expression of p‐Smad3 and nuclear translocation, ultimately promoting fibrotic protein expression and the fibrotic response.

### 3.7 A novel METTL3 TCM monomer inhibitor, isoforsythiaside, exhibits anti‐renal fibrosis potential in vitro and in vivo

**FIGURE 7:**
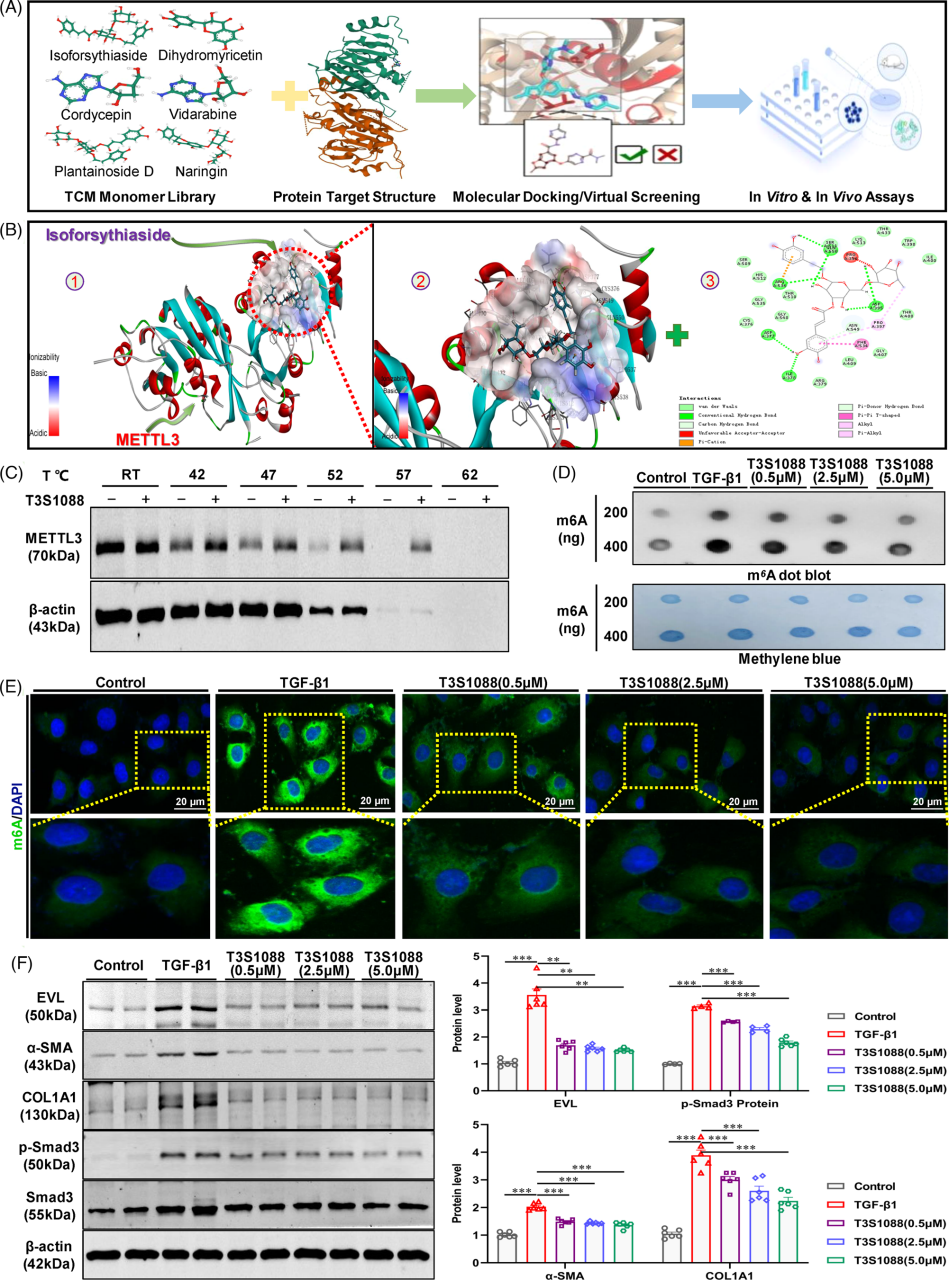
In vitro antifibrotic potential of isoforsythiaside, a novel methyltransferase‐like 3 (METTL3) traditional Chinese medicine (TCM) monomer inhibitor. (A) Schematic diagram of the virtual screening‐based strategy for exploring anti‐renal fibrosis TCM monomers targeting METTL3. (B) Molecular docking of isoforsythiaside to the catalytic core of METTL3. (C) Cellular thermal shift assay (CETSA) analyses showing the stabilisation of METTL3 in vitro with or without isoforsythiaside treatment. (D) Dot blot analysis showed that isoforsythiaside treatment reduced the *N* ^6^‐methyladenosine (m6A) abundance in transforming growth factor‐β1 (TGF‐β1)‐treated HK‐2 cells. (E) Representative immunofluorescence staining showed that isoforsythiaside treatment reduced the m6A abundance in TGF‐β1‐treated HK‐2 cells (*n* = 3). Scale bar = 20 μm. (F) Western blot analyses of Ena/VASP‐like (EVL), p‐Smad3/Smad3 and fibrotic indicators in isoforsythiaside‐treated TGF‐β1‐induced HK‐2 cells (*n* = 6). Control represents untreated HK‐2 cells. Data represent the mean ± S.E.M. of at least three to four independent experiments in vitro. Statistically significant differences were determined by independent sample *t* test and one‐way analysis of variance (ANOVA) followed by Tukey's post hoc test. ^*^ *p* < .05, ^**^ *p* < .01 and ^***^ *p* < .001.

Based on the above findings, we next explored potential therapeutic drugs that target METTL3 for the treatment of renal fibrosis. A computer‐assisted drug screening strategy was used to identify novel METTL3 inhibitors, which were subsequently evaluated for their antienzymatic activity and antifibrotic effects (Figure 7A). Of the top 20 compounds screened from the TCM monomer compound library (L6810, 2688 compounds), three TCM monomers, including T5796 (plantainoside D), T3S1088 (isoforsythiaside) and T2993 (cordycepin), showed a certain degree of antifibrotic potential (Figures S6, S7 and S8A). To identify the most promising TCM monomer therein, multiple experiments were carried out. Western blot and dot blot assays showed no significant effect of the three TCM monomers on the protein expression of METTL3, whereas isoforsythiaside had a better ability to reduce m6A modification than the other two monomers (Figure S8B,C). Coupled with the immunofluorescence staining of m6A, isoforsythiaside was the most promising TCM monomer with antifibrotic potential in our study (Figures 7E and S8D–F). The molecular structure of isoforsythiaside is shown in Figure S8A, and molecular docking analysis supported a potential interaction between isoforsythiaside and METTL3 (Figure 7B), which was confirmed by CETSA. METTL3 was denatured at a temperature range from room temperature to 62°C with or without isoforsythiaside treatment, suggesting that isoforsythiaside caused remarkable thermal translocation of METTL3 protein in HK‐2 cells. These results suggested that isoforsythiaside can bind directly to the METTL3 protein (Figure 7C). Dot blot assays revealed that isoforsythiaside (.5, 2.5 and 5.0 μM) differentially reduced the m6A modification levels in TGF‐β1‐ and H/R‐treated HK‐2 cells (Figures 7D and S9A). We next evaluated the effect of isoforsythiaside on the viability of HK‐2 cells. The 3‐(4,5‐Dimethyl‐2‐Thiazolyl)‐2,5‐Diphenyl Tetrazolium Bromide assay showed that isoforsythiaside at concentrations less than 12.5 μM had insignificant cytotoxic effects on HK‐2 cells (Figure S6). Isoforsythiaside (.5, 2.5 and 5.0 μM) alleviated TGF‐β1‐ and H/R‐induced fibrotic responses of HK‐2 cells in a concentration‐dependent manner (Figures 7F, S8H and S9E), and similar effects also occurred in the inhibition of EVL protein (Figures 7F, S8G and S9E). Immunofluorescence staining revealed that different concentrations of isoforsythiaside reduced the overactivated m6A modification in HK‐2 cells treated with stimuli (Figures 7E and S8D).

**FIGURE 8:**
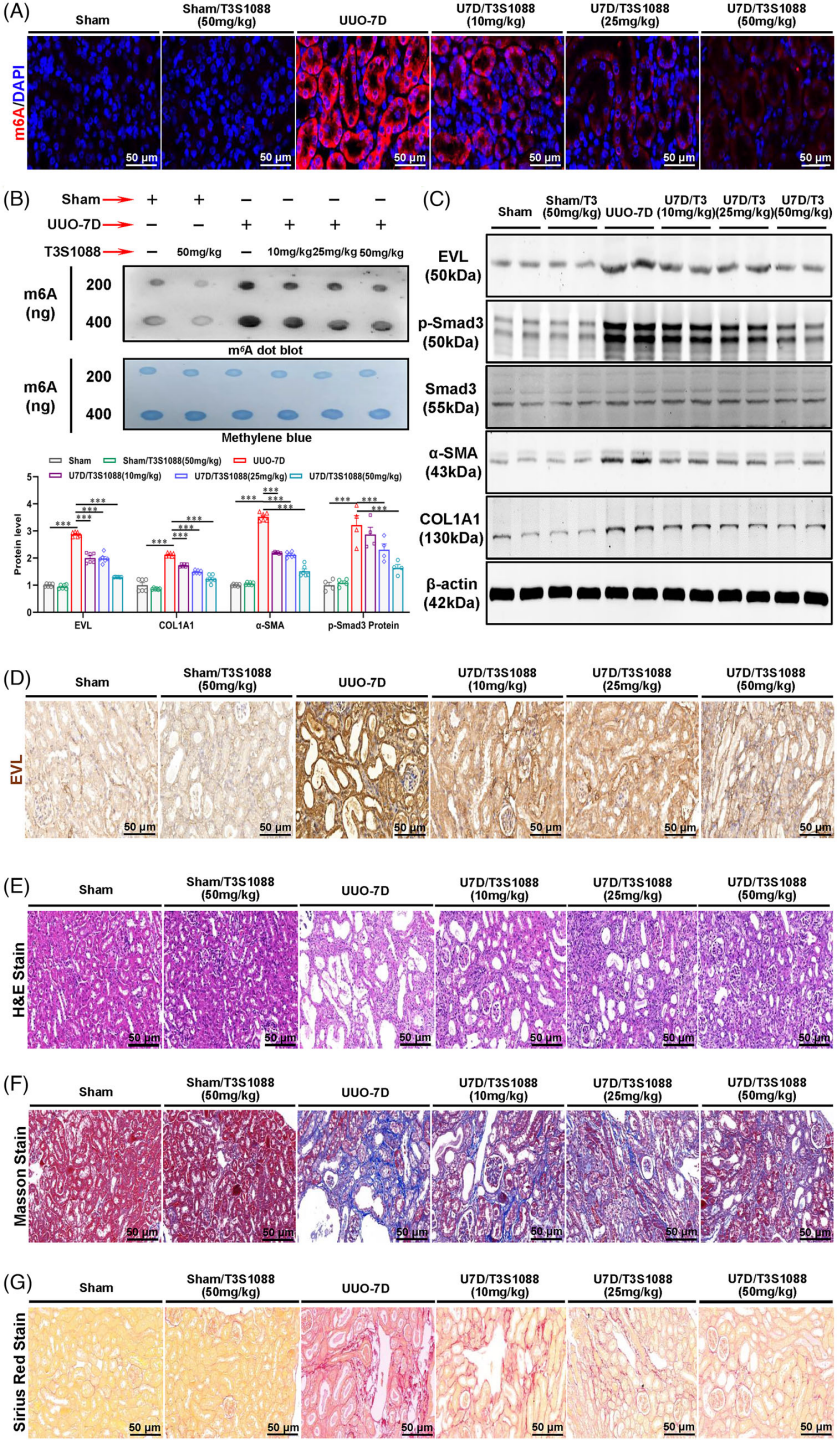
In vivo anti‐renal fibrosis potential of isoforsythiaside. (A) Immunofluorescence staining analyses of the effect of isoforsythiaside on *N* ^6^‐methyladenosine (m6A) modification in renal tissues of unilateral ureteral obstruction (UUO) mice (*n* = 3). Scale bar = 50 μm. (B) Dot blot assay showed that isoforsythiaside treatment reduced the m6A abundance in renal tissues of UUO mice. (C) Western blot analyses of Ena/VASP‐like (EVL), p‐Smad3/Smad3 and fibrotic indicators in UUO‐induced renal fibrosis kidneys treated with isoforsythiaside (*n* = 6/4). (D) Immunohistochemical analyses of the effect of isoforsythiaside on EVL expression in kidney tissues of UUO mice (*n* = 3). Scale bar = 50 μm. (E) Haematoxylin & eosin (H&E) staining analyses of the effect of isoforsythiaside on renal fibrosis in UUO mice (*n* = 3). Scale bar = 50 μm. (F) Masson staining analyses of the effect of isoforsythiaside on renal fibrosis in UUO mice (*n* = 3). Scale bar = 50 μm. (G) Sirius Red staining analyses of the effect of isoforsythiaside on renal fibrosis in UUO mice (*n* = 3). Scale bar = 50 μm. Scale bar = 50 μm. Sham represents mice subjected to sham operation. Data represent the mean ± S.E.M. of at least six mice in vivo. Statistically significant differences were determined by independent sample *t* test and one‐way analysis of variance (ANOVA) followed by Tukey's post hoc test. ^*^ *p* < .05, ^**^ *p* < .01 and ^***^ *p* < .001.

Subsequently, we evaluated the antifibrotic effect of isoforsythiaside in mouse models induced by UUO and I/R. The mice were pretreated with isoforsythiaside (10, 25 and 50 mg/kg) 24 h prior to model establishment. As shown in Figures 8A,B and S9B,C, immunofluorescence staining revealed that m6A levels in fibrotic kidney tissues significantly decreased with isoforsythiaside treatment. Immediately afterward, we found that isoforsythiaside treatment inhibited the protein levels of EVL, p‐Smad3 and fibrotic indicators in a dose‐dependent manner (Figures 8C,D and S9D,F). Moreover, the in vitro antifibrotic effect of isoforsythiaside was confirmed by H&E, Masson and Sirius Red staining (Figures 8E–G and S9G). Together, these results showed that isoforsythiaside treatment has a good effect in preventing renal fibrosis and kidney damage both in vitro and in vivo.

## 4 DISCUSSION

m6A methylation represents the most prevalent and enriched RNA epigenetic modification in mRNA, and its nonnegligible function in multiple diseases has been gradually clarified.^13^ Some recent studies have examined the relationship between m6A modification and kidney disease progression; however, they have mostly focused on AKI and renal carcinoma, while related investigations of renal fibrosis are lacking.^17^, ^19^, ^27^ In the present study, we revealed compelling evidence linking upregulated METTL3 and overactivated m6A modifications closely to renal fibrosis. Elevated METTL3 facilitated the m6A modification of EVL mRNA and promoted renal fibrosis by modulating TGF‐β1/Smad signal transduction. Accordingly, we identified a novel METTL3 TCM monomer inhibitor, isoforsythiaside, and demonstrated that it significantly inhibited the METTL3/EVL m6A axis, thereby preventing renal fibrosis. The aforementioned results collectively revealed that the METTL3/EVL m6A axis is a potential therapeutic target for renal fibrosis.

Initially, we emphasised the characteristics and potential functions of METTL3 and m6A modifications in renal fibrosis. As renal fibrosis is a common pathogenesis and pathological course in multiple CKDs, increasing evidence supports that dysfunction of renal TECs robustly contributes to renal fibrosis progression.^28^, ^29^ Hence, TECs should not only be considered a critical target of kidney injury but also a significant promoter and advocate of renal fibrosis. Therefore, targeting TECs to influence renal fibrosis may be a promising strategy for renal fibrosis prevention as well as CKD and AKI‐to‐CKD management.^30^ Unfortunately, specific therapeutic strategies are still lacking, indicating that more investigations are required to deepen the exploration. Currently, many explorations of the functions and mechanisms of m6A modifications in fibrotic diseases are underway. In liver fibrosis, ALKBH5 inhibits the mitochondrial fission, proliferation and migration of hematopoietic stem cells in a YTH domain family protein 1 (YTHDF1)‐dependent manner by reducing the m6A modification of Drp1, which provides a valuable strategy for liver fibrosis diagnosis and therapy.^31^ In bleomycin‐treated mice, upregulated METTL3 has been identified to enhance the m6A modification of KCNH6 mRNA in a YTHDF1‐dependent manner to promote its translation and expression, which evokes the phenotypic conversion of alveolar myofibroblasts and pulmonary fibrosis.^32^ These findings imply that targeted modulation of METTL3‐mediated m6A modification may also represent a breakthrough in pulmonary fibrosis management. Nonetheless, the precise roles of m6A modifications in renal fibrosis, especially the detailed mechanisms, are largely unknown and warrant focused attention. In the present study, we found that the levels of METTL3 and m6A modifications were significantly elevated in the kidney tissues of ON patients. Moreover, in both UUO and I/R‐induced renal fibrosis mice, METTL3 and corresponding m6A levels were consistently upregulated with the duration of disease, indicating a positive correlation of elevated METTL3 and m6A levels with the advancement of renal fibrosis. Moreover, METTL3 cKO in the kidneys and silencing in HK‐2 cells not only attenuated renal fibrosis induced by UUO and I/R but also reduced TGF‐β1‐ and H/R‐induced fibrotic responses. These findings collectively suggested that METTL3 functions as a profibrotic mediator in both the HK‐2 fibrotic response and kidney fibrosis. Hence, targeted regulation of METTL3 and the corresponding m6A may be a novel strategy to interfere with the HK‐2 cytofibrotic response and renal fibrosis.

During the mechanistic exploration phase, we investigated the elaborated molecular mechanisms of METTL3 in promoting renal fibrosis. We performed an integrated analysis of RNA‐seq and MeRIP‐seq results, and we observed multiple (3909 vs. 3094) genes with elevated expression levels along with upregulated m6A modifications. Moreover, a certain number of genes (2978 vs. 409) showed decreased expression levels accompanied by downregulated m6A modifications. Only nine genes showed inconsistent trends in expression and m6A modification alterations. These results demonstrated that m6A modifications positively regulate gene expression during renal fibrosis. After cross‐tabulating the sequencing results with an FC ≥2 and *p*‐value <.05 in addition to eliminating logical inaccuracies, we identified 19 potential METTL3‐modified target genes, including ACTA1, EVL, LSP1, NPY6R, PGLYRP2, TCEAL5 and VGF, which were also validated in METTL3 cKO mice. Subsequently, we performed screening with kidney tissue samples of ON patients together with TGF‐β1‐induced HK‐2 cells, which confirmed seven candidate target genes. To identify the candidate METTL3 target gene with the greatest antifibrotic potential, we silenced each of these target genes and investigated their effects on the fibrotic response in HK‐2 cells. The present data indicated that EVL silencing attenuated the TGF‐β1‐induced fibrotic response in HK‐2 cells more significantly than the other candidates. Based on these findings, EVL is a potential target gene for METTL3‐mediated m6A modifications during renal fibrosis. In addition, both the m6A modification and expression of EVL mRNA were significantly increased in multiple fibrotic cell models, while the deletion of METTL3 significantly decreased the stability of EVL mRNA and its expression, which suggested that METTL3 may affect the stability, expression and the corresponding function of EVL by enhancing its m6A modification. In many studies, reader proteins have been found to be extensively involved in the process of m6A modification mediated by methyltransferases or demethylases. In hypoxia‐induced tumour cells, ubiquitinated YTHDF2 increases its binding to m6A‐modified mRNA to promote mRNA degradation and cancer progression.^33^ During mesenchymal stem cell (MSC) senescence, IGF2BP2 increases the stability of m6A‐modified MIS12 mRNA and reverses the senescence phenotype of human MSCs.^34^ These findings suggest that reader proteins provide critical auxiliary effects in the splicing, stability and expression of mRNAs undergoing m6A modification. Based on the finding that METTL3 mediates m6A modification of EVL mRNA in renal fibrosis, promoting its stability, as well as the nonnegligible roles of IGF2BP1/2/3 in maintaining the mRNA stability where m6A modifications occur,^26^ we sought to explore whether these reader proteins impact the m6A modification of EVL. Upon silencing IGF2BP1, IGF2BP2 and IGF2BP3, we observed that IGF2BP2 knockdown was more effective in reducing the upregulated EVL mRNA levels in TGF‐β1‐treated HK‐2 cells than IGF2BP1 and IGF2BP3, indicating that IGF2BP2 may function as a contributor to the m6A modification of EVL. We also knocked down IGF2BP2 and found that it significantly reduced the development of fibrotic responses in HK‐2 cells treated with TGF‐β1 and H/R, both in terms of protein expression and mRNA expression, thus validating the involvement of IGF2BP2 in the m6A modification of EVL. In parallel, compared to IgG controls, the IGF2BP2‐specific antibody markedly enriched EVL mRNA in the RIP assay. Coincidentally, IGF2BP2 deficiency significantly diminished the stability of EVL mRNA. Taken together, the m6A modification of EVL mRNA mediated by METTL3 enhances its stability and expression, thereby affecting the fibrotic response in an IGF2BP2‐dependent manner.

After elucidating the underlying mechanism by which METTL3 modulates renal fibrosis, we investigated the function of an identified target gene of METTL3, namely, EVL, during renal fibrosis. EVL is a member of the Ena‐VASP family, which is widely distributed in multiple organs and tissues, such as lymph nodes, spleen, lung, brain, liver and kidneys.^35^, ^36^ Existing studies have revealed that EVL is widely involved in a variety of physiopathological processes, such as ruffle and stress fibre assembly, epithelial cell migration, vascular development and tumour metastasis^36^, ^37^, ^38^; however, its function in renal diseases, especially in intrinsic renal cells, is deficient and remains to be elucidated. The present study demonstrated that EVL was significantly upregulated in human fibrotic kidney tissues, UUO and I/R‐induced renal fibrosis mouse models as well as TGF‐β1‐ and H/R‐stimulated HK‐2 cells, while knockdown of EVL markedly reduced the mRNA and protein expression of fibrotic indicators, indicating that EVL may promote the fibrotic response of HK‐2 cells and renal fibrosis progression. Given the centrality of TGF‐β1/Smad3 signalling in renal fibrosis, we subsequently checked the changes in Smad3 signalling and found that silencing EVL could reduce the phosphorylation and nuclear translocation of Smad3 without any significant effect on Smad3 levels, which implies that EVL may promote renal fibrosis by modulating TGF‐β1/Smad3 signalling. Molecular docking studies suggested that EVL could bind to the Smad7 protein, which was confirmed by co‐IP assays in TGF‐β1‐treated HK‐2 cells. Furthermore, a co‐IP experiment also revealed that Smad7 binds to the TGF‐βR1 protein in addition to its binding to EVL. As a negative modulator of the TGF‐β protein superfamily, Smad7 binds to the specific E3 ubiquitin ligase Smurf2 to form a complex. The complex is subsequently recruited to TGF‐βR1 and degrades it via the proteasomal and lysosomal pathways to inhibit TGF‐β1/Smad signal transduction.^39^, ^40^ The co‐IP assay after EVL silencing showed that the binding of EVL to Smad7 protein was decreased, while the binding of Smad7 to TGF‐βR1 protein was increased. In summary, these findings indicate that multiple factors contribute to the upregulation of EVL in fibrotic kidney tissues, particularly in renal TECs. The upregulated EVL subsequently binds to the Smad7 protein to reduce the binding of the Smad7–Smurf2 complex to TGF‐βR1 and its degradation, which in turn promotes Smad3 activation, phosphorylation and even TGF‐β1/Smad3 signal transduction. The aforementioned changes ultimately promote the progression of renal fibrosis. In future studies, we intend to undertake more experiments to explore the specific mechanism of EVL binding to Smad7 that regulates TGF‐β1/Smad signal transduction in renal fibrosis.

Functional and mechanistic exploration serves as the solid foundation for interventional therapeutic studies of diseases. Although the potent functions of METTL3 have attracted increasing attention, the exploration of disease therapies targeting METTL3 is still in its infancy.^41^, ^42^ One study revealed that inhibition of METTL3 by the small molecule compound STM2457 is a promising therapeutic strategy for acute myeloid leukemia.^22^ Additionally, inhibition of METTL3 by the small molecule compound Cpd‐564 significantly reduces renal inflammation, providing a potential therapeutic strategy for AKI.^19^ Although TCMs and their monomers have powerful potential for drug development,^43^ more research remains to be done to target METTL3‐regulated fibrotic diseases. In our study, we screened and identified a novel METTL3 TCM monomer inhibitor, isoforsythiaside, from a library of TCM monomers through computer‐assisted virtual screening, cytotoxicity assays and pharmacological functional studies. In vitro studies have shown that isoforsythiaside binds to the METTL3 protein, significantly reducing overactivated m6A modifications without affecting its protein expression. Such action downregulated EVL expression in TGF‐β1‐ and H/R‐treated HK‐2 cells, which ultimately decreased the fibrotic response of HK‐2 cells. Furthermore, in vivo studies revealed that isoforsythiaside treatment significantly attenuated the overactivated m6A modifications and markedly reduced the upregulated EVL and fibrotic indicators at both the mRNA and protein levels in the renal fibrosis mouse models induced by UUO and I/R. In parallel, isoforsythiaside treatment was effective in inhibiting renal fibrosis progression and reversing renal injury to a certain degree in the aforementioned mouse models. Hence, isoforsythiaside is a prospective METTL3 inhibitor for combating renal fibrosis and has potential translational value for clinical applications.

**FIGURE 9:**
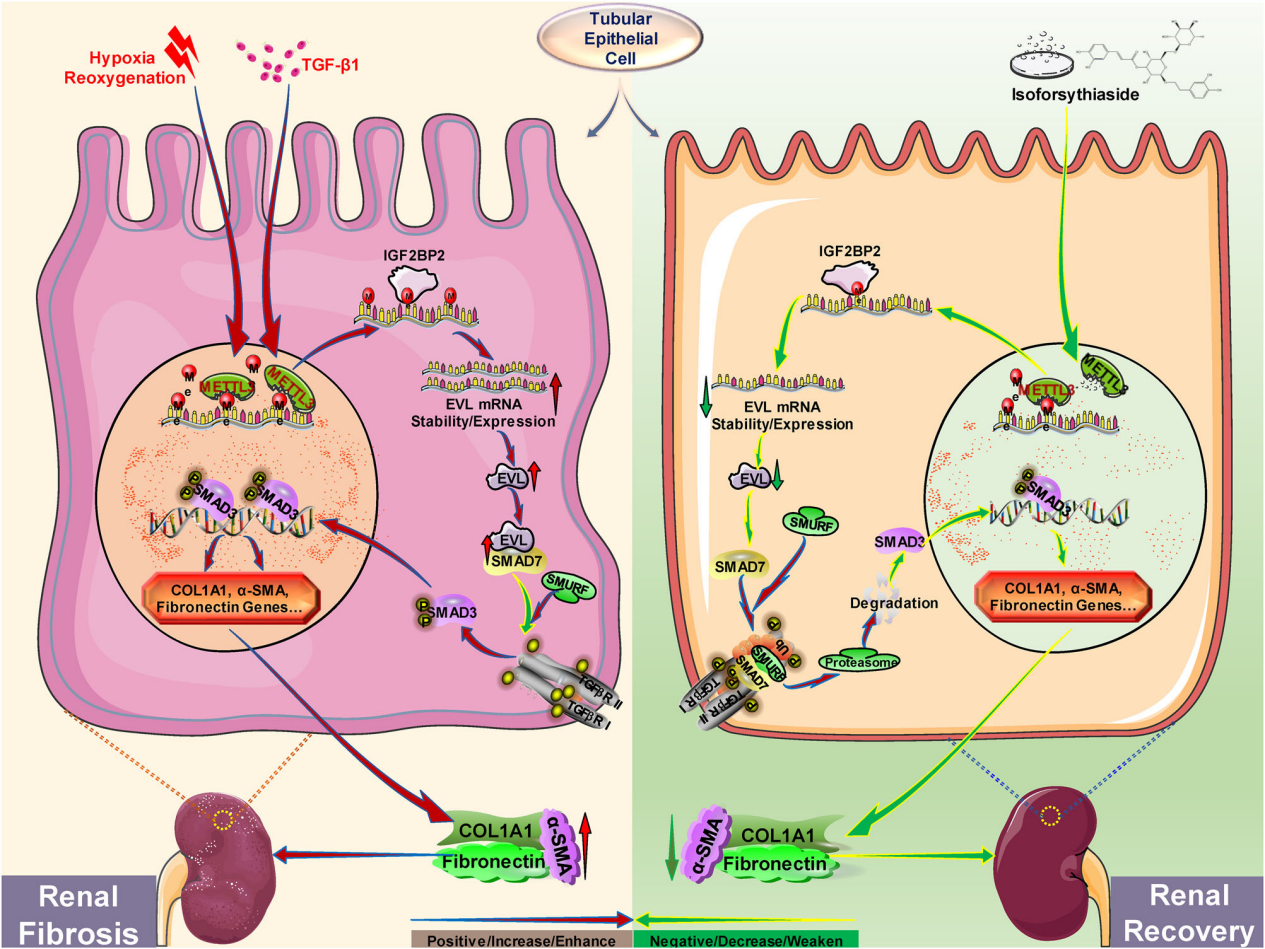
Schematic illustration of methyltransferase‐like 3 (METTL3)‐mediated Ena/VASP‐like (EVL) *N* ^6^‐methyladenosine (m6A) modification in promoting renal fibrosis and the therapeutic effect of isoforsythiaside. Upregulated METTL3 enhances the m6A modification of EVL mRNA through an insulin‐like growth factor 2 mRNA‐binding protein 2 (IGF2BP2)‐dependent mechanism, which ultimately promotes the progression of renal fibrosis. Pharmacological inhibition of overactivated METTL3 and the METTL3/EVL m6A axis suggests that isoforsythiaside is a promising anti‐renal fibrosis agent worthy of in‐depth exploration and translational research.

In summary, our findings demonstrate that multiple prefibrotic stimuli lead to significant upregulation of MELTT3 in TECs and kidney tissues, which enhances the m6A modification of EVL mRNA via an IGF2BP2‐dependent mechanism, ultimately contributing to the advancement of renal fibrosis. In parallel, the TCM monomeric isoforsythiaside based on the METTL3 structure exhibits important anti‐renal fibrosis potential through inhibition of the aforementioned process (Figure 9). The present study shed light on the currently unclear mechanisms by which m6A modifications promote renal fibrosis, and the present findings suggest that the TCM monomer isoforsythiaside, which targets the overactivated METTL3/EVL m6A axis, is a potential strategy to prevent renal fibrosis.

## CONFLICT OF INTEREST STATEMENT

The authors declare they have no conflicts of interest.

## Supporting information

Supporting Information

## Data Availability

The datasets (RNA‐seq and MeRIP‐seq) generated and/or analysed during the current study were submitted to the GEO database (GSE226505/GSE226506).
