# Supplementary material for: Genetic and pharmacological inhibition of METTL3 alleviates renal fibrosis by reducing EVL m6A modification through an IGF2BP2‐dependent mechanism
Source: Clin Transl Med. 2023 Aug 3;13(8):e1359. doi: 10.1002/ctm2.1359 (PMC10400756; doi:10.1002/ctm2.1359)
Supplement: Supplementary file 1 — Supporting Information [file CTM2-13-e1359-s001.docx]

SUPPORTING INFORMATION

**Figure S1 Characterization of renal fibrosis induced by UUO and I/R in mice and changes in regulators and m6A modifications.**

**
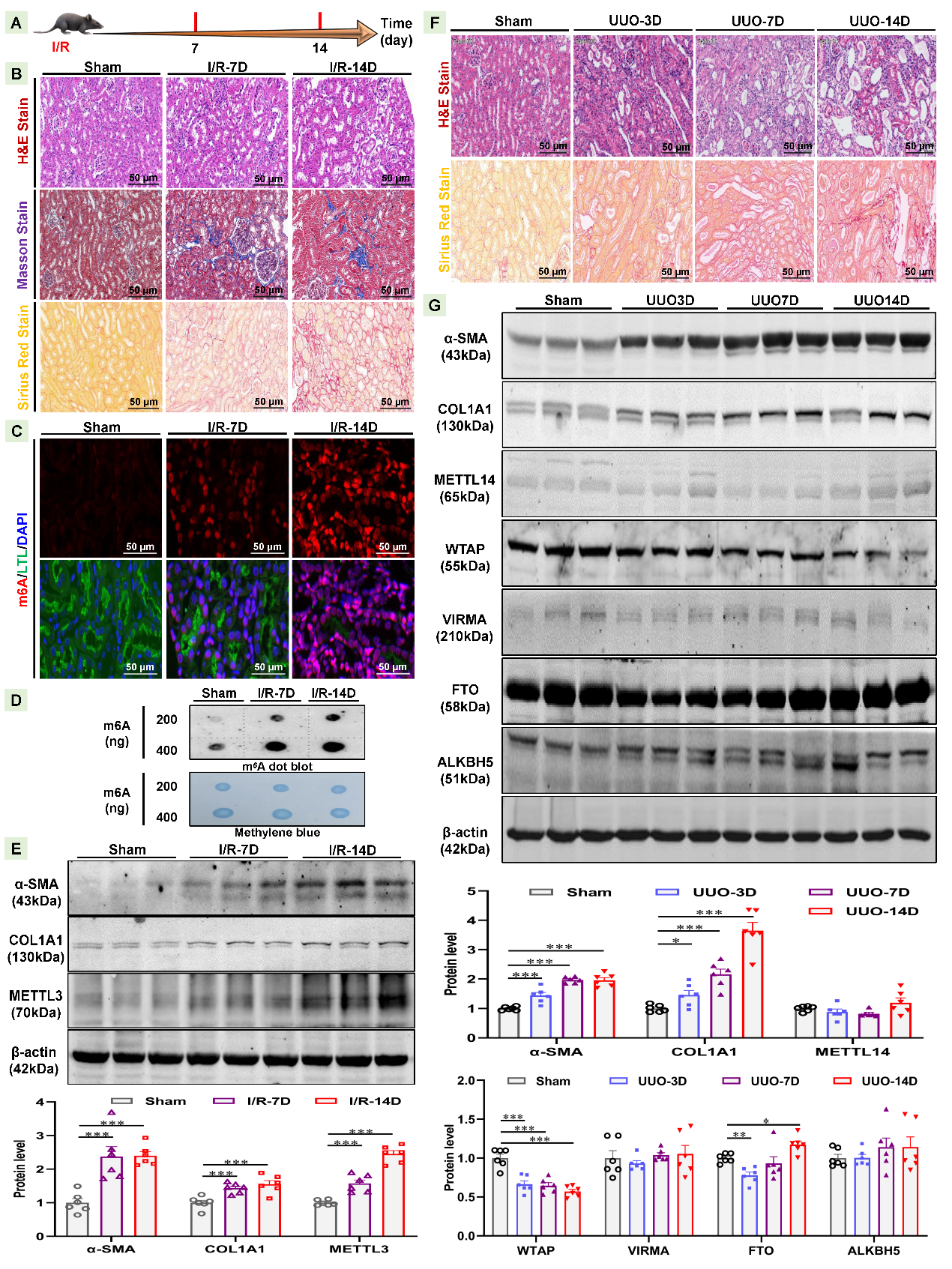
**(A) Schematic diagram of the I/R-induced mouse model of renal fibrosis. (B) Representative H&E, Masson and Sirius Red staining of kidney tissue in I/R mouse models (*n*=6). Scale bar = 50 μm. (C) Representative immunofluorescence staining of m6A and LTL in kidney tissues of I/R mice (*n*=3). LTL was used to label the proximal tubule. Scale bar = 50 μm. (D) Dot blot assay showed that I/R treatment increases m6A abundance in renal tissues. (E) Protein levels of METTL3 and fibrotic indicators in the renal tissue of I/R mice (*n*=6). (F) Representative H&E and Sirius Red staining of kidney tissue in I/R mouse models (*n*=3). Scale bar = 50 μm. (G) Protein levels of m6A regulators and fibrotic indicators in the renal tissue of I/R mice (*n*=6).

Sham represents mice subjected to sham operation; Control represents renal cell carcinoma paracellular tissue or untreated HK-2 cells. Data represent the mean ± S.E.M. of at least 6 mice *in vivo*. Statistically significant differences were determined by independent sample *t* test and one-way ANOVA followed by Tukey’s post hoc test. **P* < 0.05, ***P* < 0.01, ****P* < 0.001.

**Figure S2 Relationship of the changes in fibrotic indicators and METTL3 with m6A levels in kidney tissues of ON patients.**


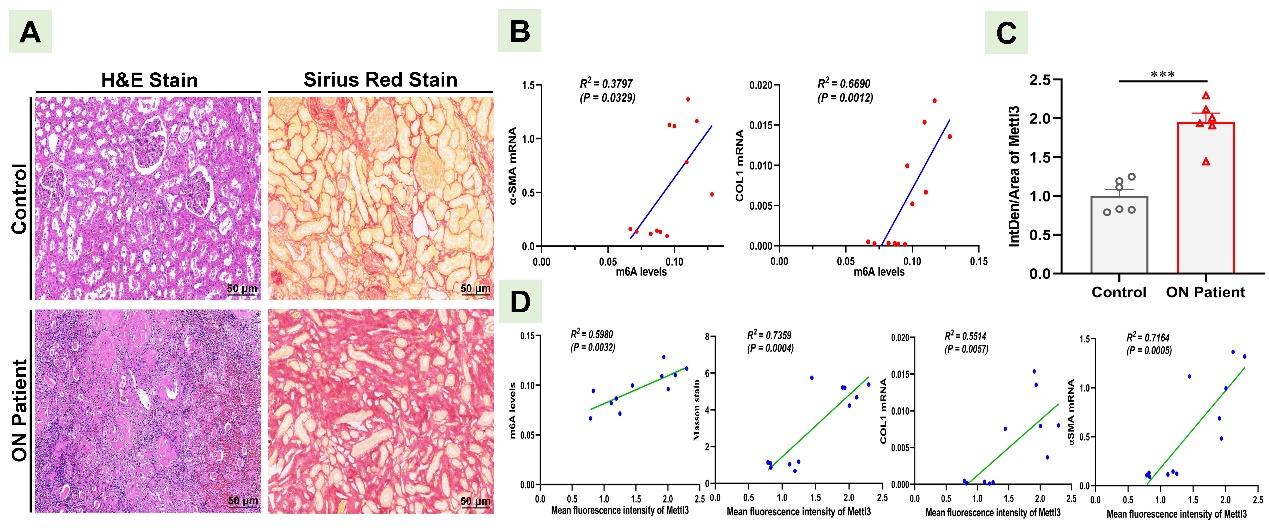
(A-B) Representative H&E and Sirius Red staining of kidney tissue in ON patients (*n*=6). Scale bar = 50 μm. (C) Pearson’s correlation analyses of the correlation between m6A levels and mRNA levels of fibrosis indicators in ON patients. (D) Quantitative analysis of METTL3 immunofluorescence staining in ON patients (*n*=6). (E) Pearson’s correlation analysis for the correlation between METTL3 and m6A levels, Masson staining score and mRNA levels of fibrosis indicators in ON patients.

Control represents renal cell carcinoma paracellular tissue. Data represent the mean ± S.E.M. of 6 patients. Statistically significant differences were determined by independent sample *t* test and one-way ANOVA followed by Tukey’s post hoc test. **P* < 0.05, ***P* < 0.01, ****P* < 0.001.

**Figure S3 Changes in METTL3 in fibrotic renal tissues and HK-2 cells and screening of downstream target genes.**


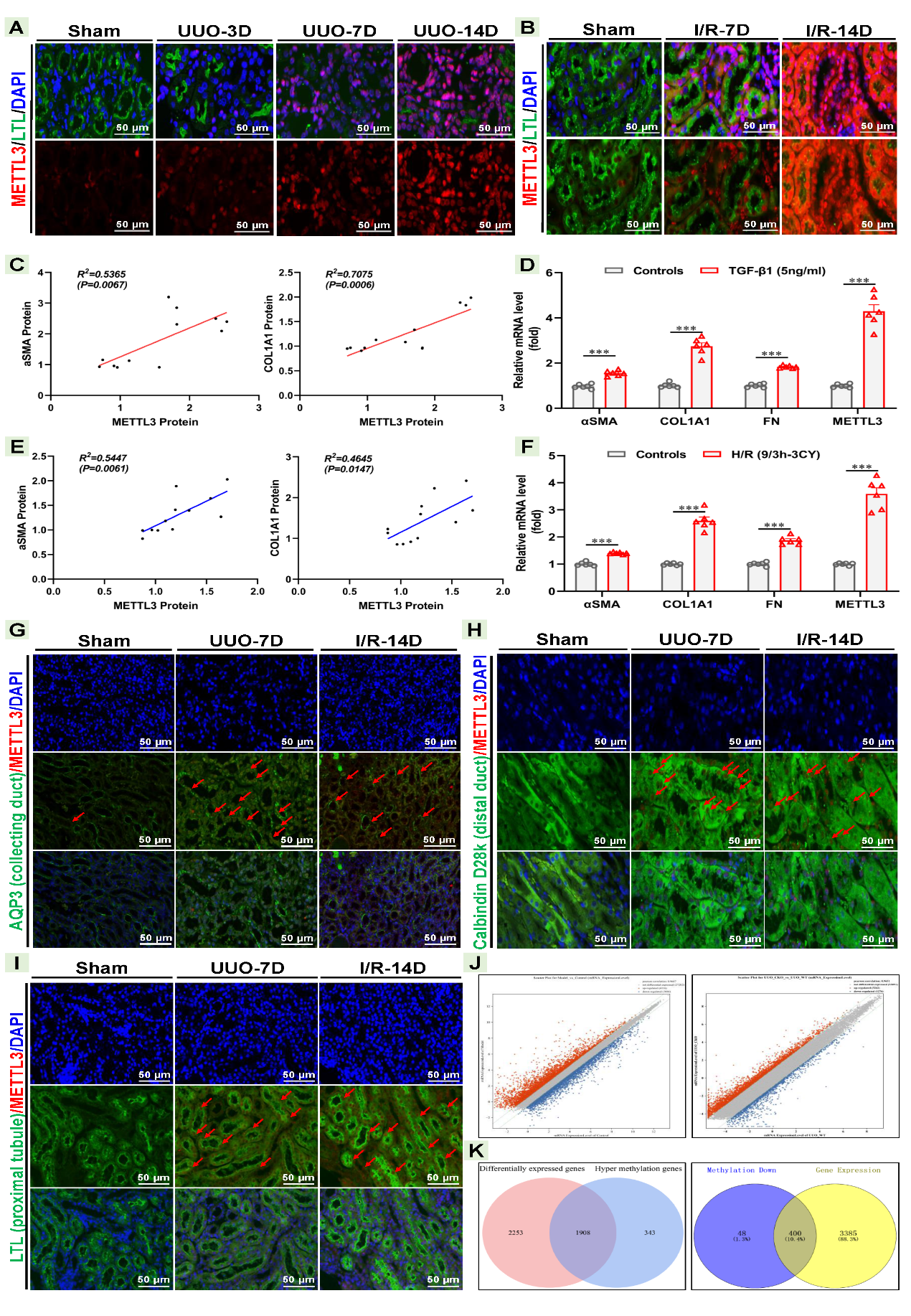
(A-B) Representative immunofluorescence staining of METTL3 and LTL in kidney tissues of UUO and I/R mice. LTL was used to label the proximal tubule (*n*=3). Scale bar = 50 μm. (C-F) mRNA levels of METTL3 and fibrosis indicators as well as Pearson’s correlation analysis for the relationship between METTL3 and fibrosis indicators in TGF-β1-and H/R-treated HK-2 cells (*n*=6). (G-I) Representative immunofluorescence staining for METTL3 with AQP3, Calbindin D28K and LTL in kidney tissues of UUO and I/R mice, respectively. AQP3 was used to label the collecting duct. Calbindin D28K was used to label the distal duct. LTL was used to label the proximal tubule. Scale bar = 50 μm. (J) Scatter plot of the mRNA expression profile in kidney tissues of UUO mice and METTL3 cKO mice. (K) Venn diagram analysis of the difference in the mRNA expression profile in kidney tissues of UUO mice and METTL3 cKO mice.

Sham represents mice subjected to sham operation; Control represents untreated HK-2 cells. Data represent the mean ± S.E.M. of at least 3 independent experiments *in vitro* and *in vivo*. Statistically significant differences were determined by independent sample *t* test and one-way ANOVA followed by Tukey’s post hoc test. **P* < 0.05, ***P* < 0.01, ****P* < 0.001.

**Figure S4 EVL is a potential target gene for METTL3-mediated m6A modification in renal fibrosis.**


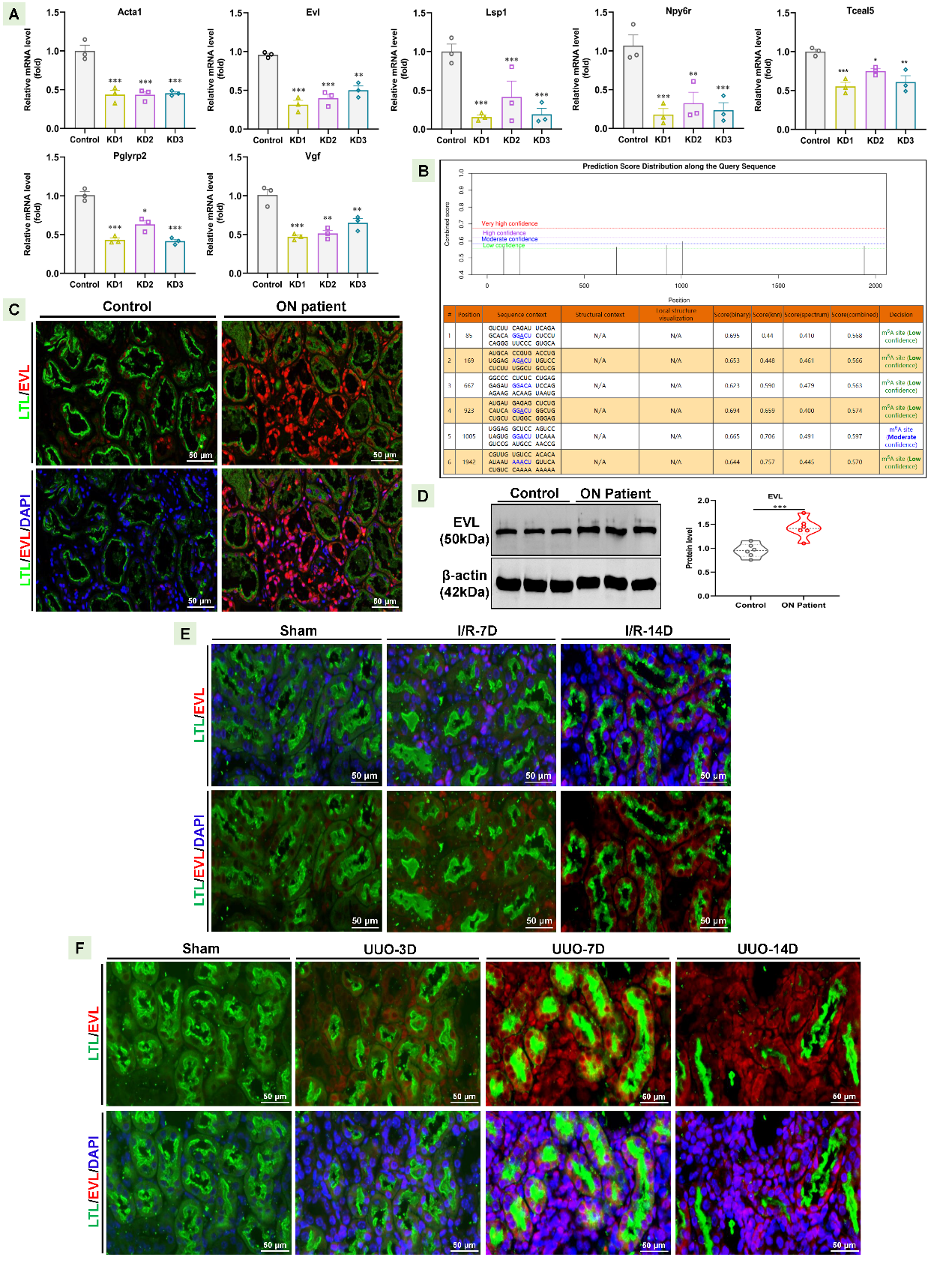
 (A) Screening for suitable ACTA1, EVL, LSP1, NPY6R, PGLYRP2, TCEAL5 and VGF siRNAs by qRT‒PCR (*n*=3). (B) Prediction of m6A modification site profiles of EVL mRNAs using the SRAMP database (www.cuilab.cn/sramp). (C) Representative immunofluorescence staining of EVL and LTL in renal tissues of ON patients (*n*=3). LTL was used to stain the proximal tubule. Scale bar = 50 μm. (D) Protein levels of EVL in renal tissues of ON patients (*n*=6). (E-F) Representative immunofluorescence staining of EVL and LTL in renal tissues of UUO and I/R-mouse models (*n*=3). Scale bar = 50 μm.

Sham represents mice subjected to sham operation; Control represents renal cell carcinoma paracellular tissue or untreated HK-2 cells. Data represent the mean ± S.E.M. of at least 3 independent experiments *in vitro* and 6 mice *in vivo*. Statistically significant differences were determined by independent sample *t* test and one-way ANOVA followed by Tukey’s post hoc test. **P* < 0.05, ***P* < 0.01, ****P* < 0.001.

**Figure S5 Silencing of EVL attenuates the fibrotic response of H/R-treated HK-2 cells probably via a Smad7/TGF-β1/Smad3 mechanism.**


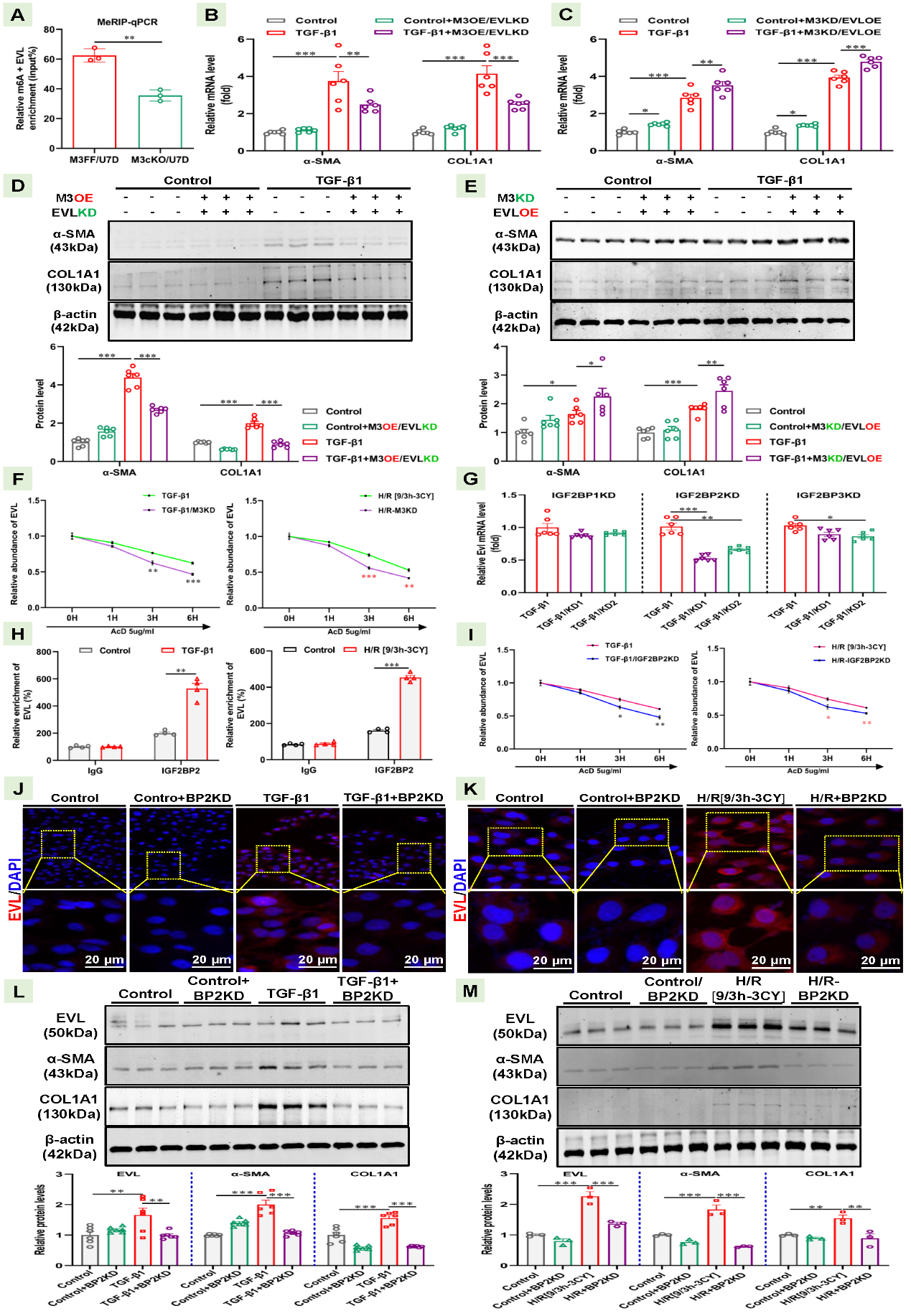
(A) Characterization of changes in EVL and METTL3 proteins in TGF-β1-treated HK-2 cells (*n*=3). (B) Screening for suitable IGF2BP1, IGF2BP2 and IGF2BP3 siRNA by qRT‒PCR (*n*=6). (C) qRT‒PCR analyses of fibrotic indicators in H/R-treated HK-2 cells with EVL knockdown (*n*=6). (D) Western blot analyses of the fibrotic indicators, p-Smad3 and Smad3 in H/R-treated HK-2 cells with EVL knockdown (*n*=3). (E) Representative immunofluorescence staining analyses of fibrotic indicators in H/R-treated HK-2 cells with EVL knockdown (*n*=3). Scale bar = 20 μm. (F) Representative immunofluorescence staining analyses of p-Smad3 in H/R-treated HK-2 cells with EVL knockdown (*n*=3). Scale bar = 20 μm.

Control represents untreated HK-2 cells. Data represent the mean ± S.E.M. of at least 3-4 independent experiments *in vitro*. Statistically significant differences were determined by independent sample *t* test and one-way ANOVA followed by Tukey’s post hoc test. **P* < 0.05, ***P* < 0.01, ****P* < 0.001.

**Figure S6 CCK-8 assay analyses of the cytotoxicity of potential TCM monomer compounds in HK-2 cells.**


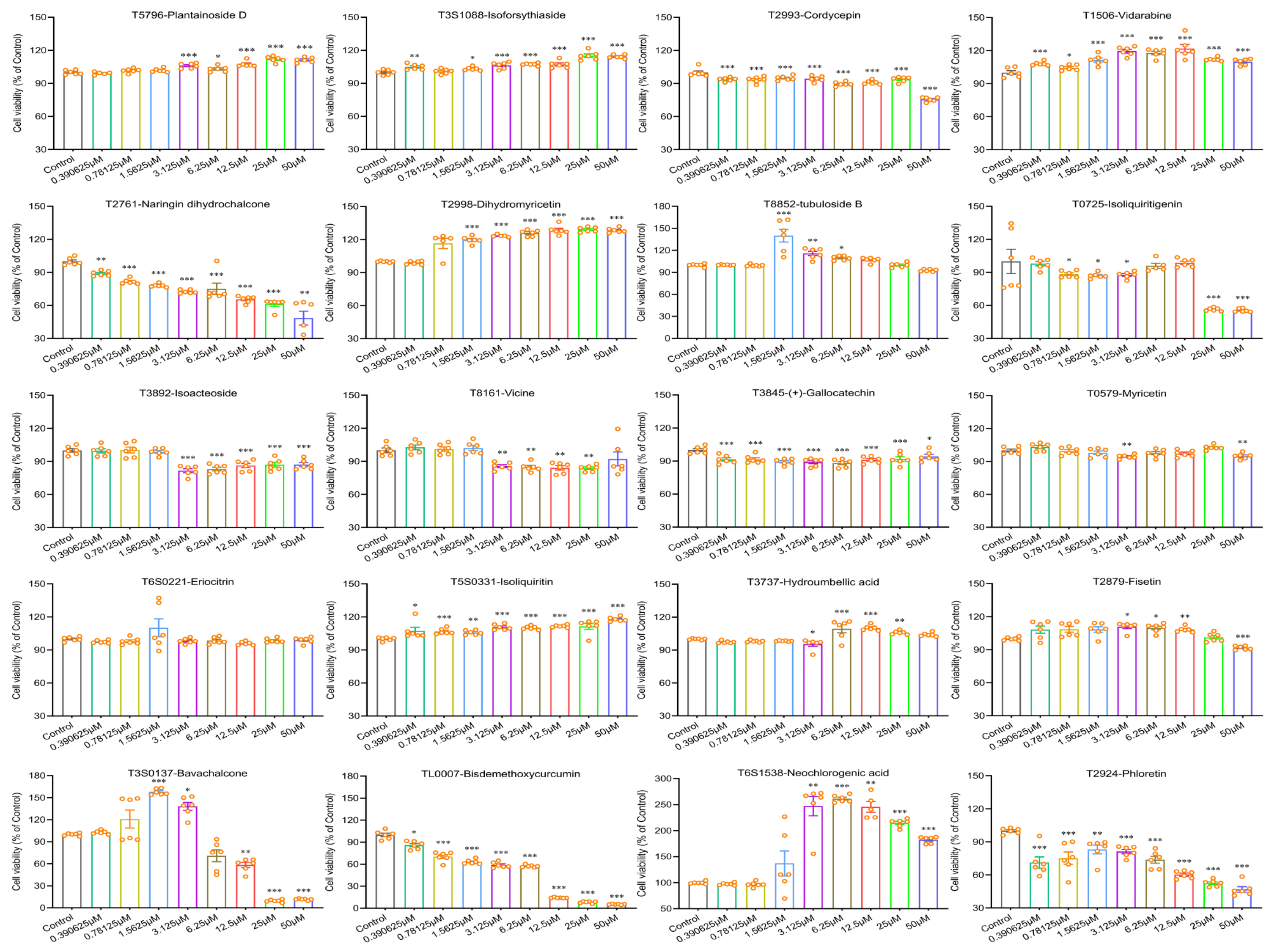
The effect of different concentrations of the top 20 TCM monomers on the viability of HK-2 cells was determined by a CCK-8 assay. Control represents untreated HK-2 cells. Data represent the mean ± S.E.M. of at least 5-6 independent experiments. Statistically significant differences were determined by independent sample *t* test and one-way ANOVA followed by Tukey’s post hoc test. **P* < 0.05, ***P* < 0.01, ****P* < 0.001.

**Figure S7** [**High-content imaging**](https://www.moleculardevices.com/products/cellular-imaging-systems#High-Content-Imaging) **analyses of the antifibrotic effect of potential TCM monomer compounds.**


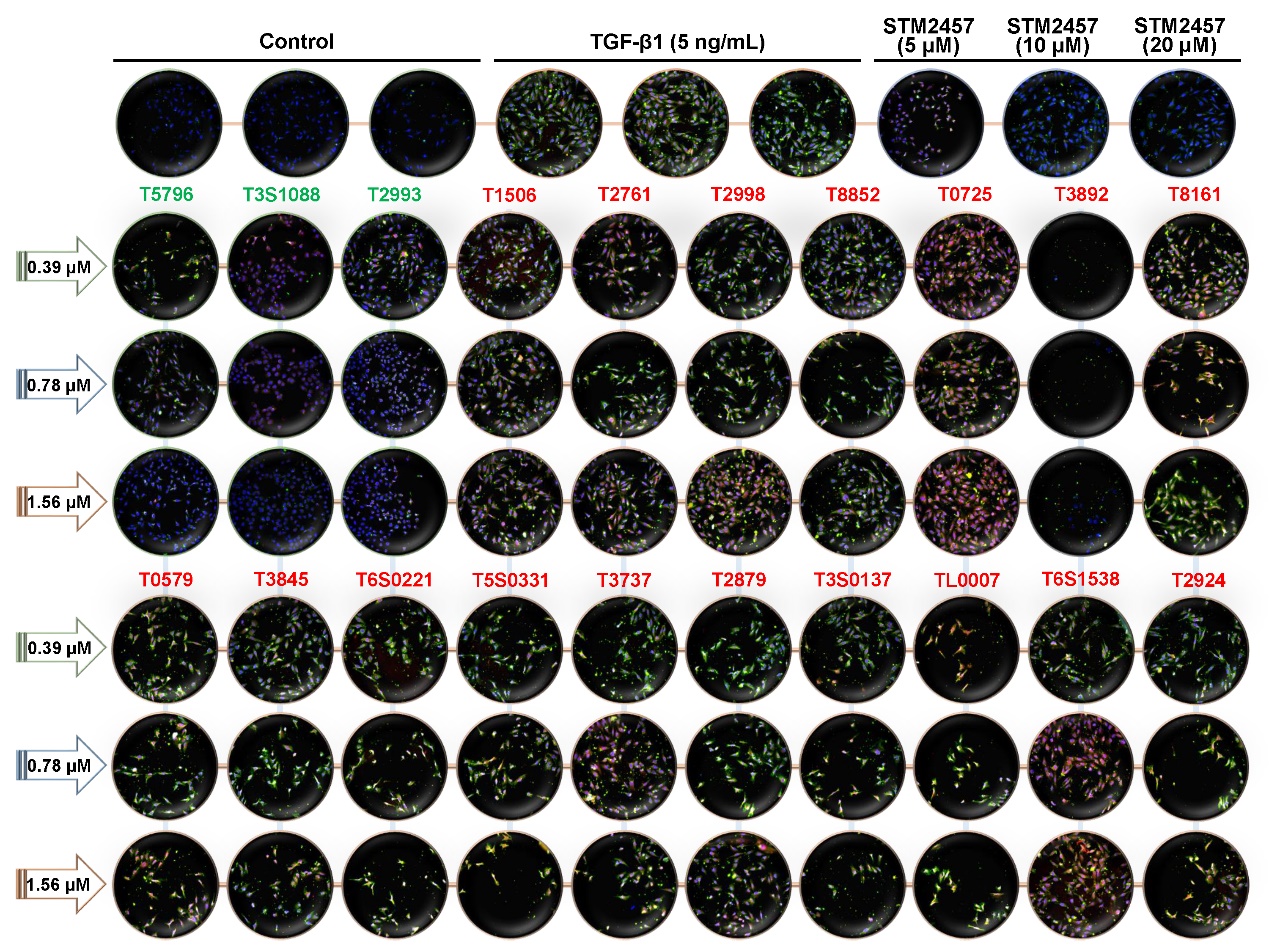
The effect of different concentrations of the top 20 TCM monomer compounds on the protein expression of fibrotic indicators in TGF-β1-treated HK-2 cells was determined by a [high-content imaging](https://www.moleculardevices.com/products/cellular-imaging-systems#High-Content-Imaging) system. Control represents untreated HK-2 cells.

**Figure S8 Effects of three TCM monomers with antifibrotic potential on METTL3-mediated m6A modification.**


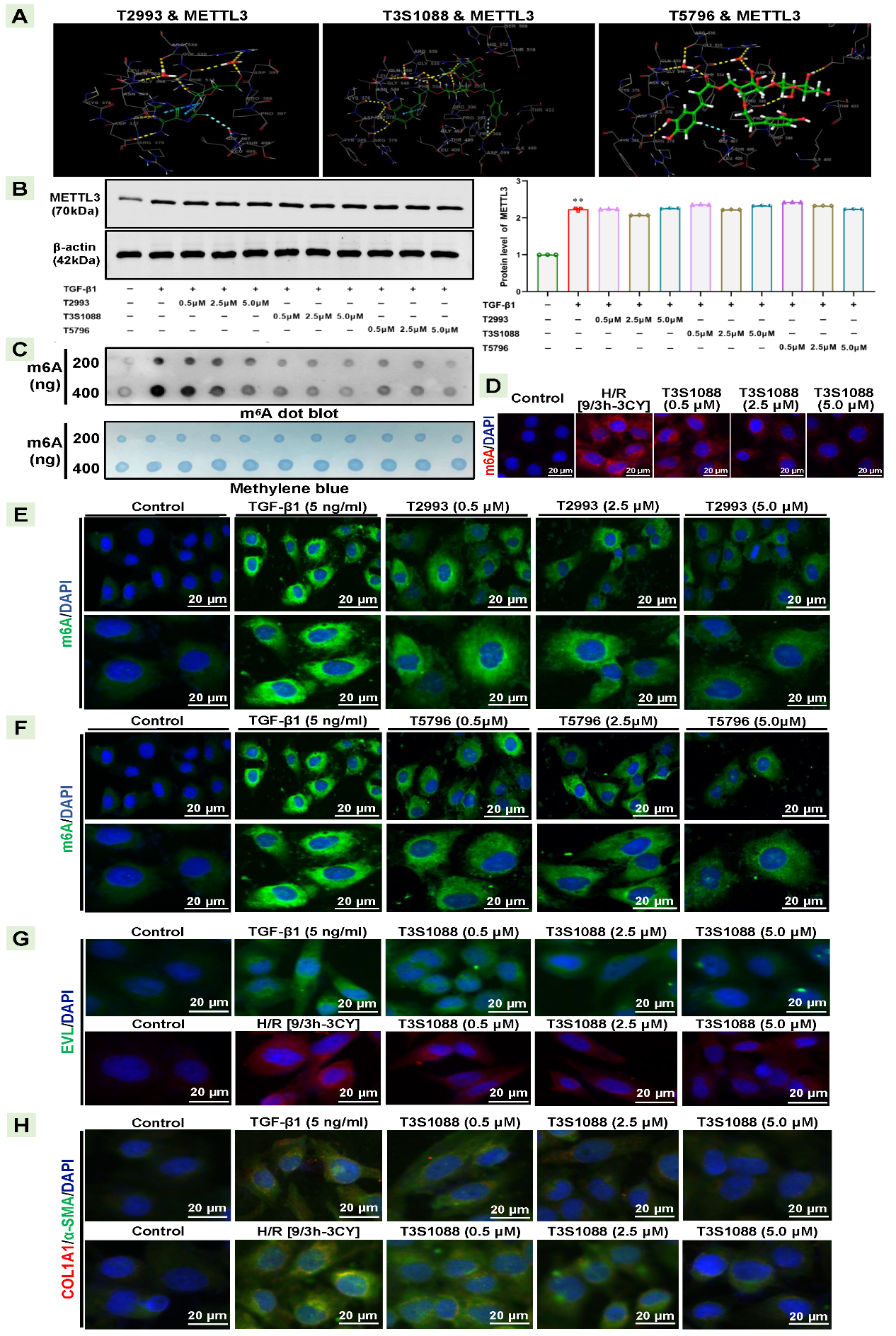
(A) Molecular docking of the three potential TCM monomers with METTL3. (B) Inhibition of METTL3 protein levels after treatment with the three potential TCM monomers in TGF-β1-treated HK-2 cells (*n*=3). (C) Dot blot assay detecting the effects of the three potential TCM monomers on m6A abundance in TGF-β1-treated HK-2 cells. (D) Immunofluorescence staining shows the inhibition of m6A modification after TCM monomer (T3S1088) treatment in H/R-treated HK-2 cells (*n*=3). Scale bar = 20 μm. (E) Immunofluorescence staining analyses of the inhibition of m6A modification after TCM monomer (T2993) treatment in TGF-β1-treated HK-2 cells (*n*=3). Scale bar = 20 μm. (F) Immunofluorescence staining shows the inhibition of m6A modification after TCM monomer (T5796) treatment in TGF-β1-treated HK-2 cells (*n*=3). Scale bar = 20 μm. (G) Immunofluorescence staining shows the changes in fibrotic indicators after T3S1088 treatment in TGF-β1- and H/R-treated HK-2 cells (*n*=3). Scale bar = 20 μm. (H) Immunofluorescence staining analyses of the changes in EVL after T3S1088 treatment in TGF-β1- and H/R-treated HK-2 cells (*n*=3). Scale bar = 20 μm.

Control represents untreated HK-2 cells. Data represent the mean ± S.E.M. of at least 3 independent experiments *in vitro*. Statistically significant differences were determined by independent sample *t* test and one-way ANOVA followed by Tukey’s post hoc test. **P* < 0.05, ***P* < 0.01, ****P* < 0.001.

**Figure S9 Isoforsythiaside exhibits anti-renal fibrosis potential by inhibiting METTL3-mediated m6A modifications of EVL mRNA.**


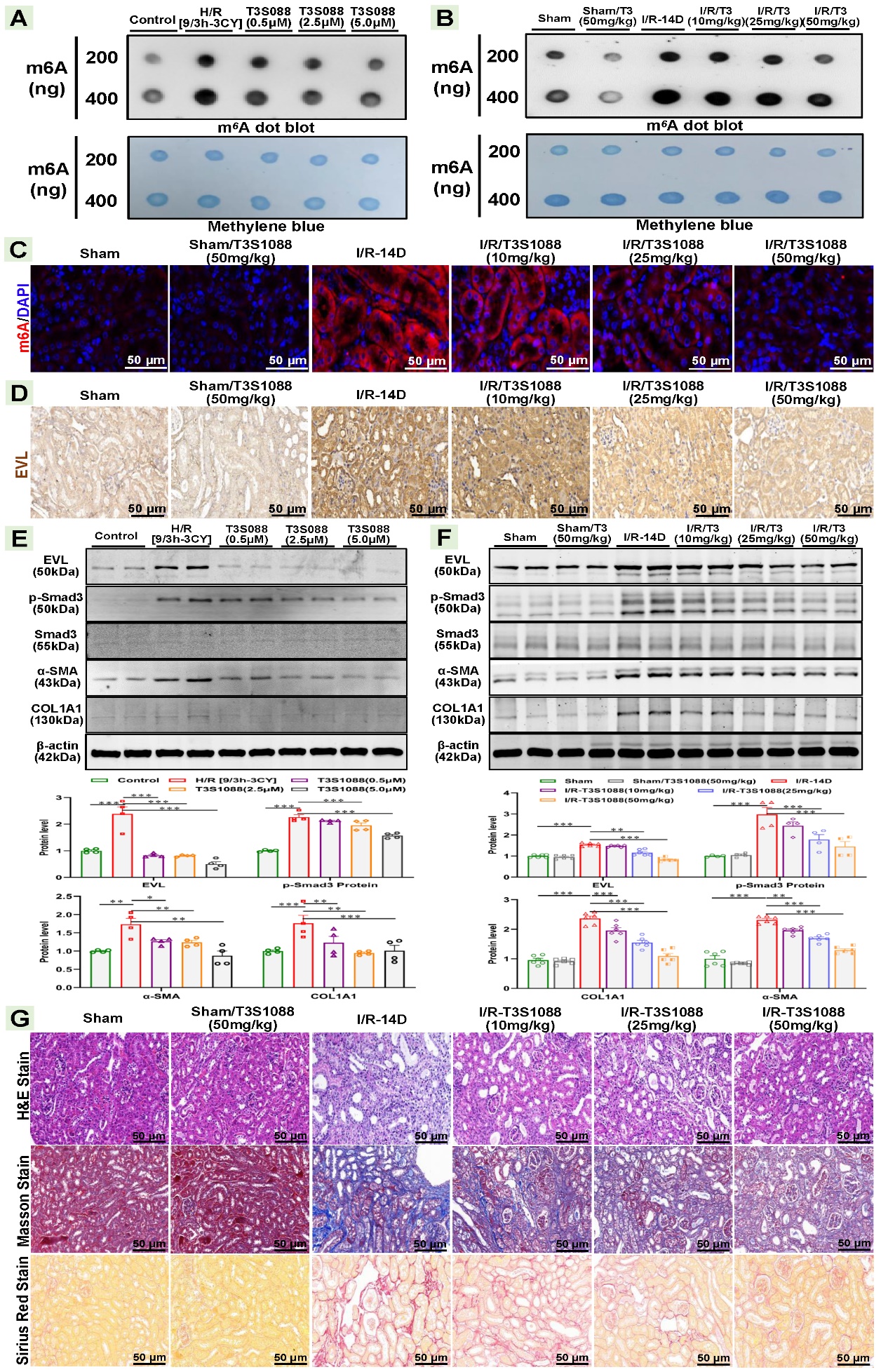
(A-B) Dot blot assay showed that isoforsythiaside treatment reduced the m6A abundance in H/R-treated HK-2 cells and renal tissues of I/R-treated mice with renal fibrosis. (C) Immunofluorescence staining of the effect of isoforsythiaside on m6A modification in kidney tissues of I/R mice (*n*=3). Scale bars = 50 μm. (D) Immunohistochemical analyses of the effect of isoforsythiaside on EVL expression in kidney tissues of I/R mice (*n*=3). Scale bars = 50 μm. (E) Western blot analyses of EVL, P-Smad3/Smad3 and fibrotic indicators in isoforsythiaside-treated H/R-induced HK-2 cells (*n*=4). (F) Western blot analyses of EVL, P-Smad3/Smad3 and fibrotic indicators in isoforsythiaside-treated I/R-induced fibrotic renal tissues (*n*=6). (G) Representative H&E, Masson and Sirius Red staining of the effect of isoforsythiaside on renal fibrosis in I/R mouse models (*n*=3). Scale bar = 50 μm.

Sham represents mice subjected to sham operation; Control represents untreated HK-2 cells. Data represent the mean ± S.E.M. of at least 3-4 independent experiments *in vitro* and 6 mice *in vivo*. Statistically significant differences were determined by independent sample *t* test and one-way ANOVA followed by Tukey’s post hoc test. **P* < 0.05, ***P* < 0.01, ****P* < 0.001.

**Table S1 Main reagents, chemicals, antibodies and softwares involved in this study.**

| **Terms** | **Names** | **Sources** | **Identifier** |
| --- | --- | --- | --- |
| **Reagent** | Recombinant Human TGF-β1 | PeproTech | Cat # 100-21C |
|  | STM2457 | Topscience | Cat # T9060 |
|  | Isoforsythiaside | Topscience | [Cat # T3S1088](https://www.tsbiochem.com/compound/isoforsythiaside) |
|  | TCM monomer | Topscience | N/A |
| **Chemicals** | Phosphatase Inhibitor Cocktail | APExBIO Technology | Cat # K1015 |
|  | Lipofectamine 2000 | Invitrogen | Cat # 11668019 |
|  | RIPA Lysis Buffer | Beyotime | Cat # P0013B |
|  | Penicillin/Streptomycin | Beyotime | Cat # C0222 |
|  | NP-40 Lysis Buffer | Beyotime | Cat # P0013F |
|  | Triton X-100 | Solarbio | Cat # T8200 |
|  | TRIzol Reagent | Invitrogen | Cat # A33250 |
|  | NT Nitrocellucose Transfer Membrane | Pall Corporation | Cat # 66485 |
|  | Actinomycin D | Sigma-Aldrich | Cat # 50-76-0 |
|  | Bovine Serum Albumin | Sigma-Aldrich | Cat # 9048-46-8 |
|  | Protein A+G-Agarose | Bioworld Technology | Cat # BD0045 |
|  | EpiQuik M6A RNA Methylation Quantification Kit | Epigentek | Cat # P-9008 |
|  | RNA Immunoprecipitation (RIP) Kit | BersinBio Biotechnology | Cat # Bes5101 |
| **Antibodies** | anti-m6A antibody | Abcam | Cat # ab284130 |
|  | anti-METTL3 antibody | Cell Signaling Technology | Cat # 86132 |
|  | anti-METTL14 antibody | Cell Signaling Technology | Cat # 48699 |
|  | anti-WTAP antibody | Cell Signaling Technology | Cat # 41934 |
|  | anti-VIRMA antibody | Cell Signaling Technology | Cat # 88358 |
|  | anti-FTO antibody | Proteintech | Cat # 27226-1-AP |
|  | anti-ALKBH5 antibody | Proteintech | Cat # 16837-1-AP |
|  | anti-IGF2BP2 antibody | Proteintech | Cat # 11601-1-AP |
|  | anti-EVL antibody | Proteintech | Cat # 13484-1-AP |
|  | anti-Smad7 antibody | Proteintech | Cat # 25840-1-AP |
|  | anti-TGF-βR1 antibody | Affinity Biosciences | Cat # 25840-1-AP |
|  | anti-Smad3 antibody | Proteintech | Cat # 66516-1-Ig |
|  | anti-p-Smad3 antibody | Abcam | Cat # ab52903 |
|  | anti-LTL antibody | Vectorlabs | Cat # FL-1321-2 |
|  | anti-COL1A1 antibody | Proteintech | Cat # 66761-1-Ig |
|  | anti-α-SMA antibody | Proteintech | Cat # 14395-1-AP |
|  | anti-β-actin antibody | Proteintech | Cat # 20536-1-AP |
| **Softwares** | IBM SPSS Statistics v26.0 | <https://www.ibm.com/cn> | |
|  | GraphPad Prism v8.3.0 | <https://www.graphpad-prism.cn/> | |
|  | ImageJ v1.8.0 | <https://imagej.nih.gov/ij/> | |
|  | Odyssey Software v3.0 | <https://www.licor.com/bio/cn/> | |
|  | CaseViewer v2.2 | https://www.3dhistech.com/ | |
|  | TissueFAXSViewer v7.0 | https://www.tissuegnostics.cn/tissuefaxsviewer/ | |
|  | Shrodinger v11.5 | https://www.schrodinger.com/ | |

***Note:*** ALKBH5: alkB homolog 5, RNA demethylase; COL1A1: collagen type I alpha 1 chain; EVL: Enah/Vasp-like; FTO: fat mass and obesity associated; IGF2BP2: insulin like growth factor 2 mRNA binding protein 2; LTL: lotus tetragonolobus lectin; m6A: N6-methyladenosine; METTL3: methyltransferase like 3; METTL14: methyltransferase like 14; STM2457: a selective METTL3 inhibitor; TCM: traditional Chinese medicine; TGF-β1: transforming growth factor-β1; TGF-βR1: transforming growth factor-β receptor 1; VIRMA: vir-like m6A methyltransferase associated; WTAP: WT1 associated protein.
